# A Layer-Based Model for Frictional Sliding of Pillar Arrays

**DOI:** 10.1021/acs.langmuir.5c05261

**Published:** 2026-02-10

**Authors:** Jasreen Kaur, Xuemei Xiao, Preetika Karnal, Chung Yuen Hui, Anand Jagota

**Affiliations:** † Department of Chemical & Biomolecular Engineering, Lehigh University, Bethlehem, Pennsylvania 18015, United States; ‡ Department of Bioengineering, 1687 Lehigh University, Bethlehem, Pennsylvania 18015, United States; § Department of Mechanical and Aerospace Engineering, 5922 Cornell University, Ithaca, New York 14853, United States

## Abstract

Bioinspired micropatterned surfaces have been studied as a way to enhance or control interfacial mechanical properties. Here, we study friction for the relative sliding of two interdigitated pillar surfaces. The overall frictional force originates from individual pillar–pillar interactions across the interface as well as the interpillar coupling within each substrate. In this study, we develop a layer-based physical model to simulate the contact and sliding behavior of these pillar arrays. The system is modeled as comprising four layers of nodes, with uniform shear displacement applied to the top layer, while the bottom one is held fixed. Nodes in the inner two layers represent the joints between the pillars and the substrate. The model predicts shear friction force and reveals underlying deformation mechanisms at various misorientations and height overlaps, in good agreement with measured friction in sliding experiments.

## Graphical abstract

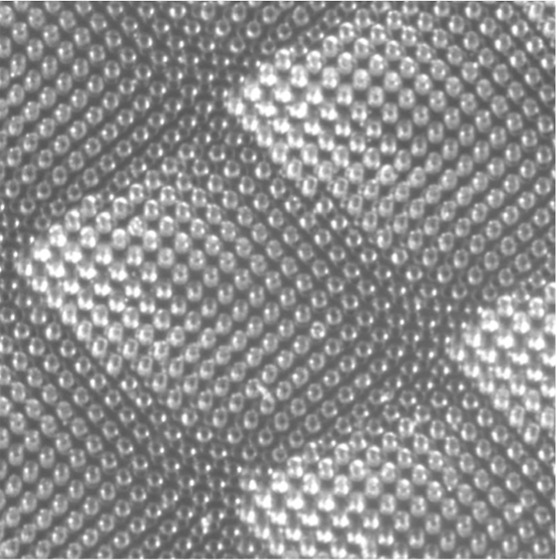

## 1 Introduction

Bioinspired structures have been recognized for their ability to enhance surface mechanical properties.^1–3^ Drawing inspiration from natural designs like the hierarchical features of a gecko’s foot^4,5^ and the head-arresting systems in^6^ dragonflies, these structures provide switchable adhesion and friction properties. Such mimicry has led to surfaces that significantly improve interfacial mechanical properties,^7–9^ enabling advancements across various fields including soft robotics^10^ and medical devices.^11^

We use bioinspired pillar structures to study how friction properties can be modulated by altering the design of near-surface architecture at the scale of a few microns. Most previous studies of biomimetic surfaces focus on single-sided patterning where only one surface is structured.^12,13^ Studies involving patterning on both interfacing surfaces are limited despite their potential to mimic more realistic biological interfaces. Bioinspired pillar-array structures allow us to study the effect of patterns on both sides and how friction can be modulated on such an interface. Shape complementary interfaces, if well-aligned, show strong enhancement of adhesion due to crack-trapping and frictional losses.^14^ Shape complementary surfaces can provide significant increase in adhesion as shown by Vajpayee et al.^15^ for rippled surfaces and Singh et al.^14^ for ridge-channel surfaces.

Guduru et al.^16^ showed that surface waviness in elastic contacts causes unstable detachment and increased energy dissipation, enhancing pull-off force and offering insight into tough adhesion mechanisms. Chen et al.^17^ also showed increase in adhesion in shape complementary pillar surfaces. Velcro^18^ serves as a classic example of how structural complementarity can be harnessed to enhance adhesion.

When two regularly patterned surfaces are brought in contact, these shape complementary structures show spontaneous eruption of Moiré patterns on the interface due to the presence of an orientation or a lattice mismatch.^19^ Moiré patterns arising from an orientation mismatch generate arrays of screw dislocations, whereas those caused by a lattice mismatch produce arrays of edge dislocations.^20–22^ When both lattice and orientation mismatches are present, the resulting Moiré patterns consist of arrays of mixed dislocations. These dislocations, acting as interfacial defects, store elastic energy that is released upon the opening of the interface. As a consequence, Singh et al.^14^ showed that the increase in the density of these dislocations in ridge/channel geometries decreases adhesion. The sliding friction of ridge/channel shape complementary interfaces is likewise strongly enhanced, but this enhancement is strongly attenuated by dislocations.^20^ The relative sliding of the interface is accommodated by the glide of dislocations. For a related but different analysis of adhesion of interdigitated pillar–pillar interfaces, see ref 23

Previous experiments on sliding friction of soft pillar interfaces have demonstrated that friction depends on the normal load, and overall friction arises due to the effect of individual pillar–pillar interaction.^21,22^ In the present study, we develop a layer-based model that simulates two arrays of pillars coming in contact and sliding relative to each other. This model is based on individual pillar deformation, allowing for pillar–pillar interactions across the interface, as well as on the same side of the interface. The model is in quantitatively good agreement with the overall friction measurements. It also provides insight into how local behavior scales to collective frictional performance, an aspect difficult to isolate experimentally. We also performed finite element (FE) simulations to corroborate our experimental results.

## 2 Experimental Materials and Methods

### 2.1 Sample Fabrication

Pillar samples are fabricated by molding the polydimethylsiloxane (PDMS) elastomer into an etched silicon master, where the pillar geometry is defined by photolithography. The PDMS precursor (silicone elastomer base) is combined with the cross-linker (curing agent, Sylgard 184 Silicone Elastomer kit, Dow Corning) in the ratio 10:1 by weight. The mixture is then poured on an etched silicon master and cured at room temperature for 2 days for PDMS to solidify. We also cure samples at different temperatures and curing times that result in slightly different periodic spacings due to thermal shrinkage. The samples used in this manuscript are, in particular, cured at 60 or 110 °C for 2 h. The cured PDMS is then carefully peeled off the silicon master. A complementary sample is fabricated in the same manner. More details on the procedure can be found in previous studies.^14,19^ Flat controls were also fabricated by using a similar procedure. Figure 1 shows a 3D view of the pillar interface obtained by scanning electron microscopy.

**Figure 1:**
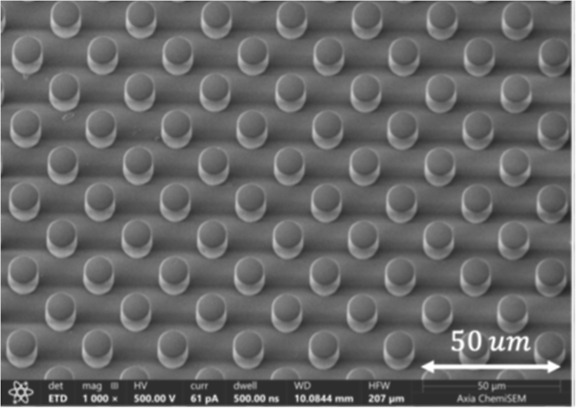
3D SEM image of micropillars arranged in a square array with a minimum interpillar spacing of 20 μm. The pillars are 10 μm in diameter and ∼16 μm in height.

### 2.2 Friction Measurement

Friction experiments are performed using a custom-built apparatus shown in Figure 2a. We mounted our samples on top and bottom stages and performed the sliding friction experiments. The typical size of a fabricated sample is 30 mm in length and a width of 10 mm on top and a smaller sample of size 4 × 4 mm for the bottom stage. More details on the experiments can be found here.^22^ The experimental setup comprises two platforms, where the test samples are secured, each equipped with a load sensor to measure forces: one for detecting lateral (shear) forces and another for vertical (normal) forces. The movement along these axes is regulated by independent vertical and horizontal motors, while an additional rotational motor adjusts the lower platform’s angle to introduce misorientation, θ. These motors are integrated with a motion control system, and the entire operation is managed by using custom-developed LabVIEW software. A high-resolution camera captures the real-time behavior of the pillars at the contact interface as sliding occurs.

**Figure 2:**
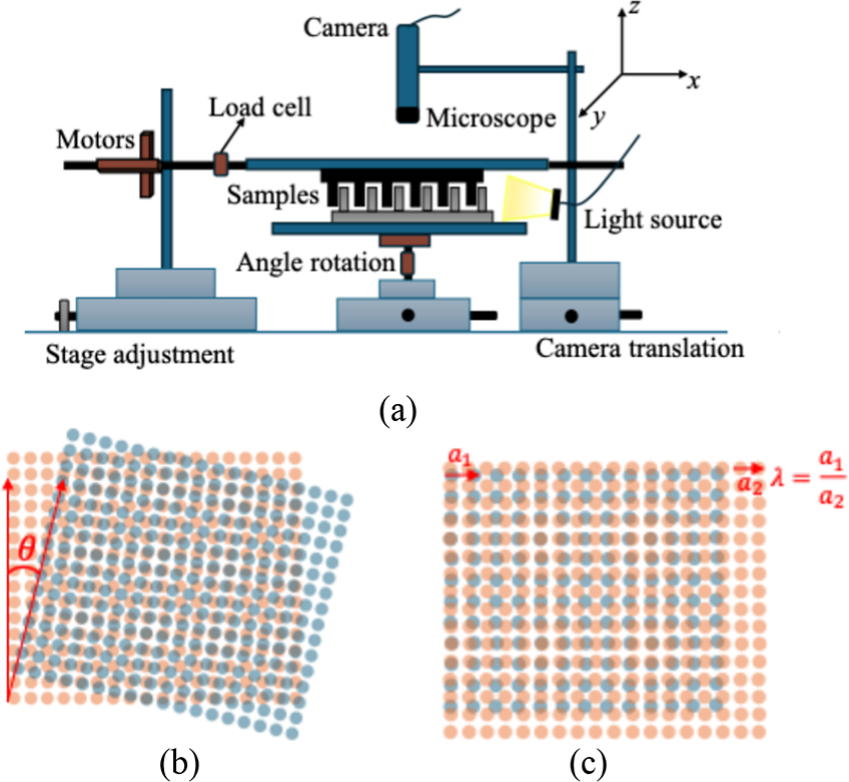
Experimental setup and definition of misorientation and lattice mismatch. (a) Custom-built tribometer for measuring sliding friction between pillar arrays. The tribometer consists of top and bottom stages where pillar samples are mounted to perform the sliding friction experiment. The bottom sample is kept smaller in comparison to the top to ensure full contact while sliding. The camera on top helps us visualize the interface. (b) Definition of misorientation, θ, which is defined as angular orientation of one sample with respect to the other. (c) Definition of lattice mismatch, $\lambda =\frac{a_{1}}{a_{2}}$ , which is defined as the ratio of lattice spacing of the two samples in contact.

Frictional sliding tests were performed at five distinct misorientation angles (θ = 0°, 5°, 15°, 30°, and 45°) and for five different normal loads, ranging from 0.075 to 0.4 N. The definitions of the types of mismatches, orientation mismatch, θ, and a lattice mismatch, λ, are illustrated in Figure 2b,c, respectively. Both patterned and flat control surfaces were tested, with each condition being repeated three times. The system can operate in two modes: maintaining a fixed normal load or controlling normal displacement. In the load-controlled mode, the applied normal force is regulated through normal displacement, which is continuously controlled by a PID controller within the LabVIEW software.

## 3 Results and Discussion

### 3.1 Moiré Pattern and Dislocations

When two micropillar samples with complementary shapes are initially brought into contact, Moiré patterns naturally emerge at the interface, as observed in prior studies.^19,20^ The periodic square arrangement of the pillars results in a structured pattern where light and dark repeating square regions appear in the interface. The size of these regions is influenced by both the angular misalignment, θ, and differences in lattice spacing, λ, which is the ratio of lattice spacing on two sides of the interface.^19^

Figure 3 shows the interfacial Moiré pattern between two pillar samples in contact with the lattice mismatch, λ = 1.006, and misorientation from θ = 0° to θ = 45°. A rotational misalignment (θ > 0° when λ = 1) leads to the formation of screw dislocations, whereas a mismatch in lattice spacing (λ ≠ 1 when θ = 0°) results in arrays of edge dislocations. When both misalignment and lattice mismatch are present (θ > 0° and λ > 1), dislocations appear with mixed screw and edge character. As illustrated in Figure 3a, when the two samples are perfectly aligned (θ = 0°, λ ≠ 1), the Moiré pattern only appears because of a lattice mismatch but are not clearly visible because the lattice mismatch is very small (λ = 1.006), leading to a large Moiré period. However, as the angular mismatch increases, the density of these patterns increases systematically, as shown in Figure 3b,c, and patterns are clearly discernible. Additional cases for λ = 1 and 1.023 are shown in Figures S1 and S3. Figure 4 shows the corresponding images from the coordinates before the sliding simulation starts in the layer-based model for λ = 1.006 and θ = 0°, 5°, and 15°. (model discussed later in Section 3.3).

**Figure 3:**
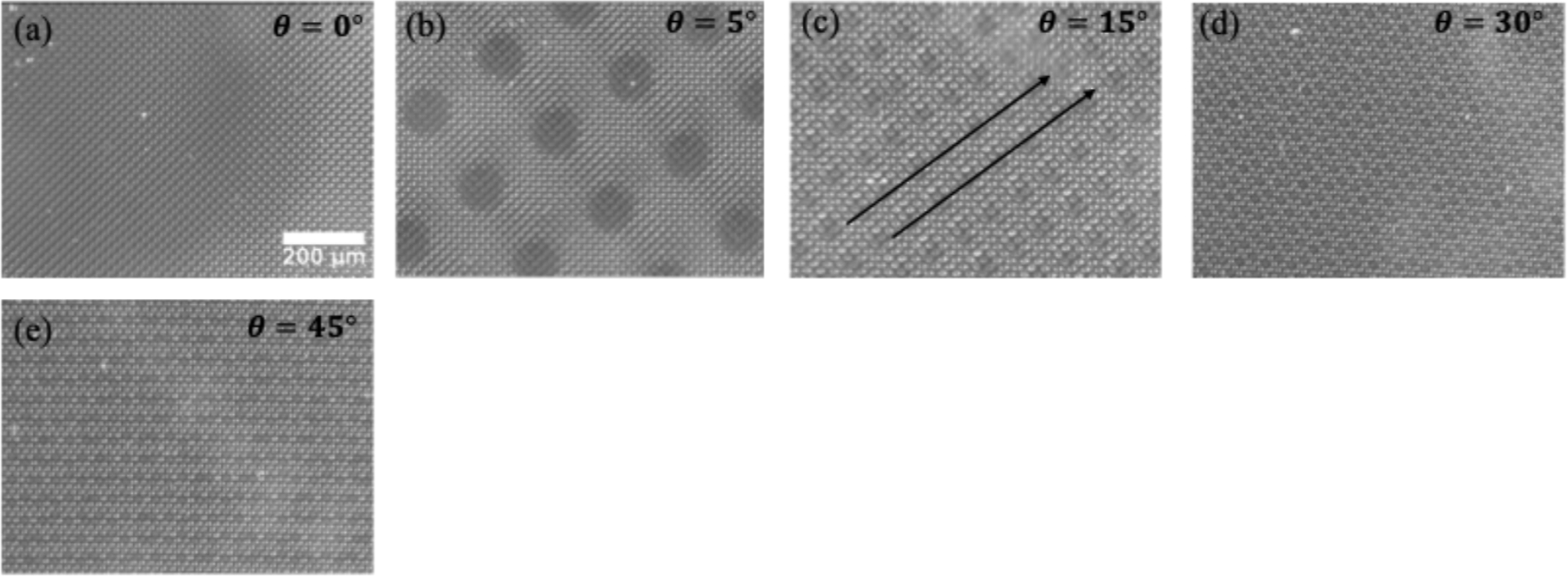
Formation of Moiré patterns and dislocations for λ = 1.00 at the interface of pillar samples on the micron scale. Appearance of the Moiré pattern on pillar structures at several misorientations, θ (a) θ = 0°, (b) θ = 5°, (c) θ = 15°, (d) θ = 30°, and (e) θ = 45°. The black lines in (c) show dislocation lines. As θ increases, the density and periodicity of the Moiré pattern become more pronounced and finer, indicating increased local mismatch and higher density of dislocation-like features.

**Figure 4:**
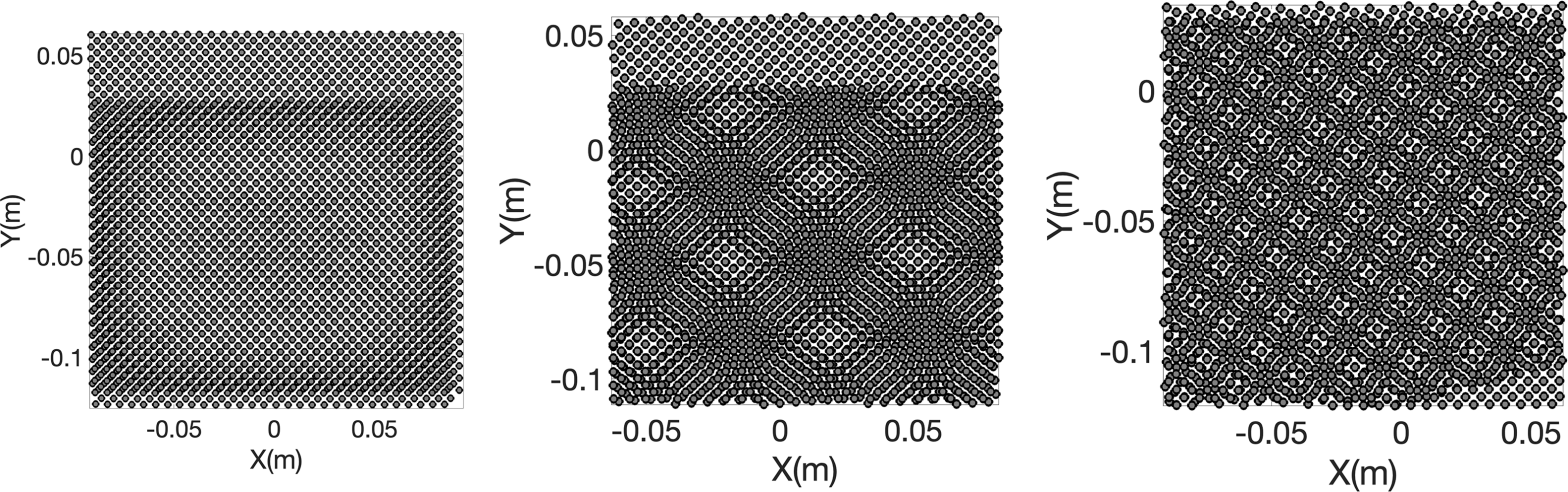
Representation of Moiré patterns from the layer-based model at angles, θ = 0° to 15° and λ = 1.006°. (a) θ = 0°, (b) θ = 5°, and (c) θ = 15°. (Compare with Figure 3a–c.)

These Moiré patterns correspond to dislocation arrays. Therefore, an increase in pattern density corresponds to a higher number of dislocations at the interface, as shown in eqs 1 and 2 in Section S1.2. We have previously demonstrated^22^ that the overall frictional force in micropillar arrays originates from the interactions between individual pillars. There, we considered the limit where pillar compliance is much larger than substrate compliance so that pillars deform independently of each other, and the total friction force can simply be calculated as the sum of forces between individual interacting pillar pairs. More generally, pillars interact with one another due to substrate elasticity. This interaction can change the friction force and displacement patterns and is included in this work.

### 3.2 Friction between Pillar Surfaces

We measured the friction between two pillar samples using a custom-built tribometer. Sliding friction experiments were conducted under different normal forces ranging from 0.075 to 0.4 N and at different misorientations, θ = 0°, 5°, 15°, 30°, and 45°. Forces thus measured were converted to stress by dividing by area of the sample ∼16 mm^2^. Figure 5a shows shear stress versus displacement for θ = 0°, λ = 1.006, at several normal stresses. It can be seen that shear stress increases with normal stress. Figure 5b shows typical friction stress versus normal stress for different misorientations in comparison with a control. The shear stress plotted in Figure 5b is the average value of the shear stress plotted in Figure 5a during steady sliding, here taken to be the region from 1 to 3 mm sliding distance. The rest of the cases from experiments for λ = 1, 1.023 are shown in Figures S2 and S4.

**Figure 5:**
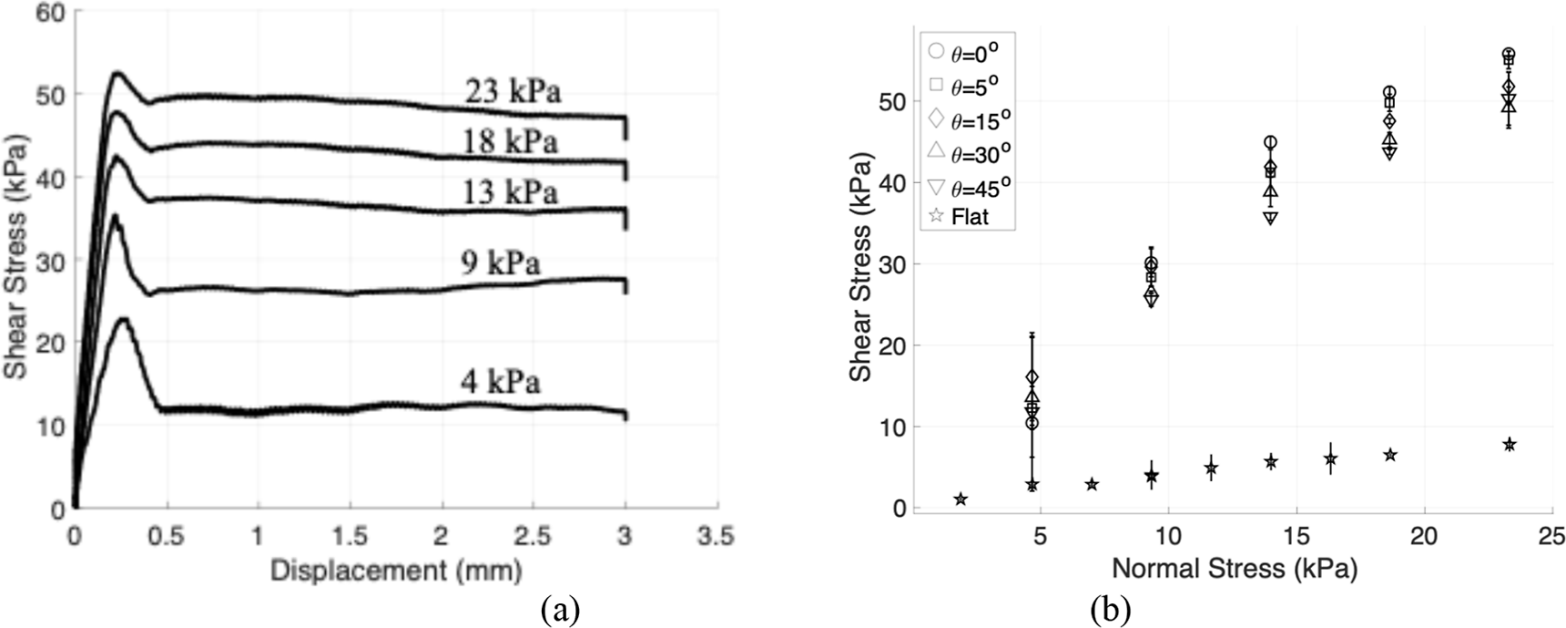
Sliding friction stress and its average value. (a) Shear stress vs displacement curves at a lattice mismatch of λ = 1.006 and misorientation angle θ = 0°, shown for multiple applied normal stresses. These results highlight how increasing normal load influences the onset and magnitude of shear stress. (b) Friction stress as a function of normal stress at the same mismatch λ = 1.006, plotted for various misorientation angles (θ = 0°, 5°, 15°, 30°, and 45°). Results are compared with those from a flat control sample showing a significant increase due to surface patterning.

In previous work, we performed single pillar-pair experiments on mm scale pillars to visualize the motion of pillars during sliding.^22^ The single pillar, fabricated at the millimeter scale, has a height of 4.8 mm and a diameter of 3 mm, maintaining the same aspect ratio as that of the micropillars. These experiments showed that overall friction force arises from individual pillar–pillar interaction. We measured the progression of sliding and bending of contacting pillar-pairs as they slid apart. (See ref 22 which shows the shear and normal force curves for single pillar-pair experiments.) These experiments were performed at several diametric overlaps ( $\overset{®}{d_{O}\;}=$ 1, 0.75, 0.5, 0.25, and 0) and various heights of contact (*h*_c_) ranging from 4.8 mm to 0.8 mm. Here, $\overset{®}{d_{O}\;}$ is normalized lateral offset and *d*_O_ refers to the lateral offset (as shown in Figure 6e) between two cylindrical pillars, with $\overset{®}{d_{O}\;}=$ 1 indicating full alignment (maximum overlap) and $\overset{®}{d_{O}\;}=0$ corresponding to incipient contact with no overlap. The height of contact, *h*_c_ (Figure 6f), represents the vertical height of the overlap between the interacting pillars. We performed quartic fits to shear force versus displacement experiments for different heights of contact. We also showed corresponding normal forces for different heights of contact with quartic fits. The parameters from these fits were then used in our simulations to model pillar interaction forces. These interpillar interaction measurements are combined with the layer-based model discussed below to calculate friction stress. This approach was necessary because the pillars are quite stocky and deformations are large. This made classical beam bending theory to model pillar–pillar interactions inapplicable to our experiments.

**Figure 6:**
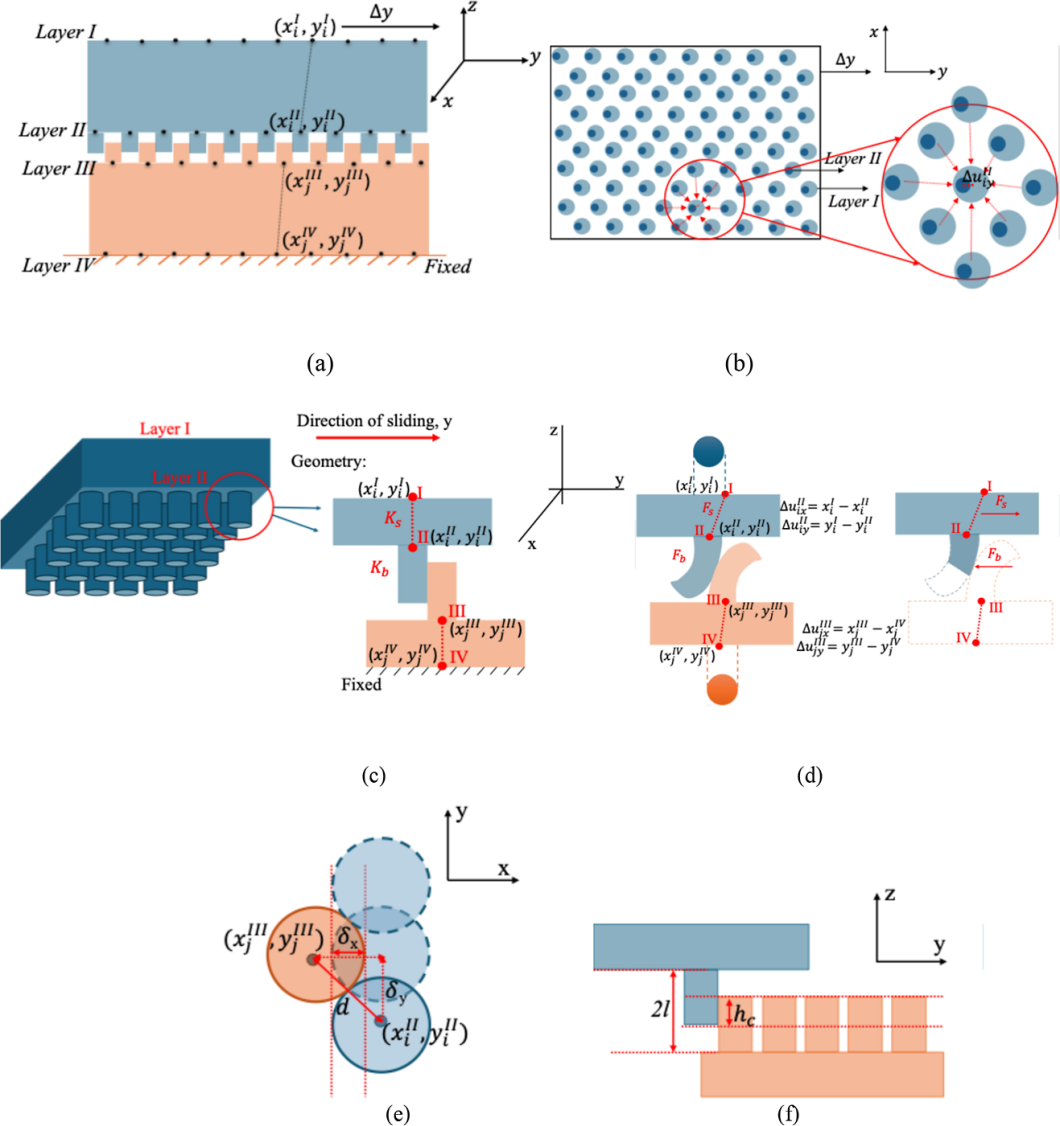
Schematic drawings of the samples and definition of variables. (a) Schematic of the cross-sectional view of pillar arrays showing pillars in contact. Defining geometry of pillar arrays in contact consisting of layers I, II, III, and IV. Nodes in the top layer, I, are given incremental displacements in the “*y*” direction. Nodes in layers II and III move because of unbalanced forces, which stops when equilibrium is achieved. The procedure is repeated incrementally. (b) Top view of layers I and II, given a uniform sliding displacement, Δ*y*, in layer I. (c) 3D rendering of the upper part of the specimen (left) showing labels for node location and a pair of pillars in contact (right) with *x*–*y* locations described at each node. (d) Pillar–pillar contact defining shear displacement in the deformed state (left). Pillar contact defining force observed on the bending pillar (right). (e) Depiction of the diametric overlap (δ_*x*_) between two pillars in contact and *d* refers to the distance between the centers of interacting pillars. δ_*y*_ refers to the relative sliding displacement in the shear direction, *y*. (f) Description of height overlap (*h*_c_) for interacting pillars and 2*L* denotes the gap between surfaces of interacting pillars.

### 3.3 Layer-Based Model

Our previous work showed that when pillar–pillar contact compliance dominates substrate compliance, the total friction force arises from summing the contributions of individual pillar interactions during sliding. To model more complex scenarios, we have developed a layer-based model, as described below. This model allows for greater flexibility and accounts for substrate compliance, and selecting variables such as pillar height, diameter, density, and substrate elasticity—parameters that are challenging to manipulate in experimental settings.

The layer-based model represents the pillar interface as a four-layer system of nodes (Figure 6a). The four layers are defined as follows: layer I corresponds to the top surface of the upper substrate, layer II represents the base of the pillars attached to the upper substrate; layer III represents the base of the pillars on the lower substrate, and layer IV corresponds to the bottom surface of the lower substrate, which is fixed.

The interface is parallel to the *x*–*y* plane. The interface bears and transmits shear load in this *x*–*y* plane and a normal load in the “*z*” direction. Figure 6a shows a cross-sectional view of the interface with a uniform shear displacement (in the *x*–*y* plane) applied to the top layer (layer I). Figure 6b provides a top view of the interface, showing nodes in layers I and II. Each node in layer II (smaller dark blue circles) lies at the center of the pillar, where it joins its substrate. For each node in layer II, there is a corresponding node in layer I (larger light blue circles). In the absence of any shear deformation, the “*x*–*y*” coordinates of a node in layer II and the corresponding node in layer I are identical. The zoomed-in region of Figure 6b highlights the relative *y* displacement between layers I and II, denoted as Δ*u*_*iy*_^II^, that arises when a motion, say Δ*y*, is applied to layer I. Figure 6c (left) is a 3D rendering of the top part of the specimen showing layers I and II. The top part interacts with a similar lower part (layers III and IV). Figure 6c (right) shows a configuration of two interacting pillars, one from each part. The *x*–*y* location of node “*i*” in layer I of the configuration is denoted by (*x*_*i*_^I^,*y*_*i*_^I^) and so-on for the other layers. Stiffness coefficients of the substrate are denoted as “*k*_s_” with additional subscripts as needed. Figure 6d (left) represents bending of pillars in contact, showing deformation in upper and lower substrates as Δ*u*_*ix*_^II^, Δ*u*_*iy*_^II^, Δ*u*_*jx*_^III^, and Δ*u*_*jy*_^III^. Figure 6d (right) shows bending of pillars in contact and substrate force and beam force as observed during bending.

Starting with an equilibrium state, we apply a uniform incremental displacement to the top layer I in the sliding or “*y*” direction. This sets up unbalanced forces on the contact layers II and III, which initiates motion of their nodes, and the system relaxes to a new equilibrium state. The net force on nodes in layers II and III has two contributions: a substrate force, *F*_s_, due to substrate deformation and a beam force, *F*_b_, due to the bending of pillars (Figure 6d). The substrate force, *F*_s_, is the restoring force exerted on nodes in contact layers (layers II and III) due to their elastic coupling to layers I and IV, respectively, and to other nodes within their own layer. The beam force arises due to pillar–pillar contact and deformation as detailed later. After equilibrium, this process is repeated with an incremental increase in displacement applied to layer I. To achieve equilibrium, for each increment of displacement of layer I, we solve the following equation of motion until the right-hand side of eq 1 is smaller than a preset value

1 $$\eta \frac{d\left(u\right)}{dt}=\left(F_{b}\right)+\left(F_{s}\right)=\left(F\right)$$

where (*F*) is the total force vector, (*F*_s_) is a vector of forces from the substrate, (*F*_b_) is a vector of forces due to bending, both functions of (*u*), which is the vector of nodal displacements, η represents a viscous damping coefficient, which modulates the rate of change of displacement, and $\frac{d\left(u\right)}{dt}$ describes the velocity of nodes. Note that the force and displacement vectors have dimensions of 2­(*N* + *M*) × 1, where *N* and *M* are the number of nodes in layers 2 and 3, respectively (i.e., we balance forces in the “*x*” and “*y*” directions on each node: see eqs S23 and S24 for details).

Equation 1 is a numerical tool to iteratively relax the system toward force balance. The left-hand side represents a damping term that gradually diminishes as the system evolves. At each increment, the solution is updated to reduce the net residual force, and equilibrium is considered achieved when the average of forces on the right-hand side reduces below a predefined fraction of a characteristic force value.

2 $$\left(F_{s}\right)+\left(F_{b}\right)=\eta \frac{d\left(u\right)}{dt}=\left(0\right)$$

This approach is particularly advantageous in handling discontinuities that arise from sudden changes in contact conditions, such as when pillar–pillar contacts break or form, where traditional quasi-static solvers may fail or exhibit numerical instability.

We next relate forces to the nodal displacements. Since the substrate is much thicker (∼1 mm) than the characteristic in-plane distances between pillars (∼20 μm), it is appropriate to approximate both the top and bottom substrates as elastic half-spaces connected to their respective pillars, each pillar being represented by a node. Consider node “*i*” in layer II (Figure 6a). Let the shear displacements of this node relative to layer I be Δ*u*_*ix*_^II^ and Δ*u*_*iy*_^II^, where the superscript denotes the layer number and the subscript indicates the node number and direction (*x* or *y*). Displacements in *x* and *y* directions on node *i* and between layers I and II are defined as

3 $$\Delta u_{ix}^{II}=x_{i}^{I}-x_{i}^{II}$$

4 $$\Delta u_{iy}^{II}=y_{i}^{I}-y_{i}^{II}$$

These shear displacements are due to the force on node *i* and a sum of influences on node *i* from all other nodes *j* on substrate II. This relationship is expressed using stiffness coefficients derived from elasticity theory for an elastic half-space (details in Section S2)­

5 $$\Delta u_{ix}^{II}=\frac{F_{si,x}^{II}}{k_{sii}}+\sum_{\begin{array}{l} j=1\\ j\neq i \end{array}}^{N}\left(\frac{F_{sj,x}^{II}}{k_{sx,ij}}+\frac{F_{sj,y}^{II}}{k_{sxy,ij}}\right)$$

6 $$\Delta u_{iy}^{II}=\frac{F_{si,y}^{II}}{k_{sii}}+\sum_{\begin{array}{l} j=1\\ j\neq i \end{array}}^{N}\left(\frac{F_{sj,x}^{II}}{k_{sxy,ij}}+\frac{F_{sj,y}^{II}}{k_{sy,ij}}\right)$$

Δ*u*_*ix*_^II^ is the total *x*-direction displacement on layer II at node *i*, where Δ*u*_*si*,*x*_^II^ is the *x*-component of the substrate force at pillar *i* of layer II, *F*_*sj*,*x*_^II^ is the substrate force at pillar *j* in the *x*-direction, *F*_*sj*,*y*_^II^ is the force in the *y*-direction at pillar *j* of layer II, and ***N*** is the total number of nodes in layer II. Similarly, Δ*u*_*iy*_^II^ is the total *y*-direction displacement on layer II at node *i*.

Substrate shear stiffness (*k*_*sii*_) relates force on pillar *i* to its contribution to displacement and is defined from ref 24

7 $$k_{sii}=\frac{16\times G\times R}{\left(2-\nu \right)}$$

where *G* is the shear modulus, *R* is the pillar radius, and *v* is Poisson’s ratio. The stiffnesses *k*_*sx*,*ij*_ and *k*_*sy*,*ij*_ are likewise derived from ref 24

8 $$k_{sx,ij}=4\pi G{\left[\frac{1}{\Delta r_{ij}^{II}}+\frac{{\left(\Delta x_{ij}^{II}\right)}^{2}}{{\left(\Delta r_{ij}^{II}\right)}^{3}}\right]}^{-1}$$

*k*_*sx*,*ij*_ captures how much *x*-force pillar *i* experiences due to a *x*-displacement at neighboring pillar *j*, where Δ*x*_*ij*_^II^ = *x*_*i*_^II^ – *x*_*j*_^II^ and $\Delta r_{ij}^{II}=\sqrt{{\left(\Delta x_{ij}^{II}\right)}^{2}+{\left(\Delta y_{ij}^{II}\right)}^{2}}$ , where Δ*r*_*ij*_^II^ is the distance between centers of pillars *i* and *j.* In the results presented later, Poisson’s ratio, for the elastomer used in experiments, ν = 0.5 and shear modulus *G* = 0.67 MPa. Similarly,

9 $$k_{sy,ij}=4\pi G{\left[\frac{1}{\left(\Delta r_{ij}^{II}\right)}+\frac{{\left(\Delta y_{ij}^{II}\right)}^{2}}{{\left(\Delta r_{ij}^{II}\right)}^{3}}\right]}^{-1}$$

A third substrate stiffness, *k*_*fxy*,*ij*_, quantifies *x*–*y* coupling

10 $$k_{sxy,ij}=4\pi G{\left[\frac{\Delta x_{ij}^{II}.\Delta y_{ij}^{II}}{{\left(\Delta r_{ij}^{II}\right)}^{3}}\right]}^{-1}$$

These stiffnesses encode how forces on the pillar base relate to its elastic deformation. The same process is followed for the lower part of the specimen with layer III playing the role of layer II and layer IV playing the role of layer I; in this case, layer IV is held fixed. Details of this derivation are described in Section S2.

These expressions can be compactly written in matrix form to describe the displacement of all of the pillars in the system. Letting (Δ*u*) be the vector of displacements and (*F*_s_) the vector of substrate forces, we write

11 $$\left(\Delta u\right)={\left[C\right]}_{s}\left(F_{s}\right),,\left(F_{s}\right)={{\left[C\right]}_{s}}^{-1}\left(\Delta u\right)$$

[*C*]_s_ is the substrate compliance matrix, consisting of self-compliances (diagonal) and interaction compliances (off-diagonal) (see eq S24). The equation above applies to layer II. A similar equation applies to any pillar *j* on layer III to calculate substrate forces on layer III defined as

12 $$\Delta u_{jx}^{III}=\frac{F_{sj,x}^{III}}{k_{sjj}}+\sum_{\begin{array}{l} k=1\\ k\neq j \end{array}}^{M}\left(\frac{F_{sk,x}^{III}}{k_{sx,jk}}+\frac{F_{sk,y}^{III}}{k_{sxy,jk}}\right)$$

13 $$\Delta u_{jy}^{III}=\frac{F_{sj,y}^{III}}{k_{sjj}}+\sum_{\begin{array}{l} k=1\\ k\neq j \end{array}}^{M}\left(\frac{F_{sk,x}^{III}}{k_{sxy,jk}}+\frac{F_{sk,y}^{III}}{k_{sy,jk}}\right)$$

where “*k*” refers to a neighboring pillar on layer III and *M* is the number of nodes on layer III. Sliding experiments and simulations are generally conducted with a smaller upper part sliding on a larger lower part and so *M* need not equal *N*.

Next, we move on to the calculation of pillar interaction forces, which occur due to the interaction between pillars on layers II and III. These forces are caused by the relative sliding motion and contact between individual pillars during bending. The magnitude of beam force depends primarily on the overlaps, δ_*x*_, δ_*y*_, and height of contact (*h*_c_) between each interacting pillar pair (as shown in Figure 6e,f). These two geometric parameters determine the extent of pillar deformation, with larger overlaps resulting in higher restoring pillar interaction forces, as described previously.^21^ To identify interacting pairs, the model calculates the center-to-center distance, *d*, between each pillar on layer II and all pillars on layer III. The pillar interaction is considered active when *d* ≤ 2*R*, where R is the pillar radius. In Figure 6e, blue circles represent the undeformed cross section of pillar *i* in the top layer (layer II), and the orange circle represents the undeformed cross-section of pillar *j* on the bottom layer (layer III). The blue circle moves in the “*y*” direction, and the lowest one represents incipient contact between pillars *i* and *j*. The next higher blue circle represents the case where pillar–pillar overlap is maximal, which we define as the diametral offset. (The two pillars will never make contact if the diametral offset exceeds the pillar diameter.) The variable δ_*y*_ is the shear displacement between the two interacting pillars in the sliding direction. This is the quantity that is fed into the empirical quartic force–displacement relation derived from single-fiber shear tests. This relation describes how much lateral force a pillar–pillar interaction generates as they slide past each other, and δ_*y*_ changes accordingly.

The model uses empirical quartic polynomial fits with coefficients (p_1_, p_2_, p_3_, and p_4_) derived from single-pillar-pair shear experiments to relate pillar interaction forces to displacements, δ_*y*_ and δ_*x*_;^21^ see Figure 6e. The pillar interaction force in the shear direction, *y*, on pillar *i* is calculated as

14 $$F_{b,y}=-\left[p_{4}{\left(\delta_{y}\right)}^{4}+p_{3}{\left(\delta_{y}\right)}^{3}+p_{2}{\left(\delta_{y}\right)}^{2}+p_{1}\left(\delta_{y}\right)\right]$$

where $\delta_{y}=-\left(\left(y_{j}^{III}-y_{i}^{II}\right)-\sqrt{{\left(2R\right)}^{2}-{\left(x_{i}^{II}-x_{j}^{III}\right)}^{2}}\right)$ . This force is turned off when δ_*x*_ > *R*, the condition under which two contacting pillars slip (see FE results in the next section).

The “*x*” force on pillar *i* is calculated as

15 $$F_{b,x}=-\left[p_{4}{\left(\delta_{x}\right)}^{4}+p_{3}{\left(\delta_{x}\right)}^{3}+p_{2}{\left(\delta_{x}\right)}^{2}+p_{1}\left(\delta_{x}\right)\right]$$

where δ_*x*_ = 2*R* – |*x*_*i*_^II^ – *x*_*j*_^III^| is the overlap in the “*x*” direction.

These fits were originally obtained from millimeter-scale single-pillar-pair experiments. To apply them at the microscale of the simulation, we rescale the displacements and forces under the assumption that stress remains scale-invariant. Specifically, if the geometric scale factor between mm-scale experiments and multipillar micron-scale experiments is α, we scale down our displacements by α and forces by α^2^. The pillars used in single-pillar experiments have a diameter of 3 mm and a height of 4.8 mm, while the microscale pillars have a diameter of 10 μm and a height of 16 μm. The geometric scaling factor α = 300. This scaling allows us to conduct simulations at the mm scale and to scale forces and displacements to the micron scale. By the same token, we can state that for a given geometry, stresses increase with shear modulus. This is tested by direct FE modeling; see Section S4.4.

The total force in eq 1, (*F*), is column vector for “*x*” and “*y*” components at each node and can be written as a concatenation of forces on nodes in layers II and III. (Nodes in layers I and IV are fixed.) For layer II, we write

16 $$\left(\begin{array}{l} F_{x}^{II}\\ F_{y}^{II} \end{array}\right)=\left(\begin{array}{l} F_{sx}^{II}\\ F_{sy}^{II} \end{array}\right)+\left(\begin{array}{l} F_{bx}\\ F_{by} \end{array}\right)$$

and for layer III

17 $$\left(\begin{array}{l} F_{x}^{III}\\ F_{y}^{III} \end{array}\right)=\left(\begin{array}{l} F_{sx}^{III}\\ F_{sy}^{III} \end{array}\right)-\left(\begin{array}{l} F_{bx}\\ F_{by} \end{array}\right)$$

Once forces at nodes in layers II and III are known, for each increment, we solve eq 1 using simple Euler forward integration in time until equilibrium is achieved. We validate our numerical approach by comparing with independently calculated solutions for some simple cases.

### 3.4 Simulation Results

To compute predicted friction forces, we chose a system of ∼1700 pillars sliding over ∼3200 pillars, a system large enough to capture interfacial dislocation arrays and result in steady-state overall shear stress while sliding. This lattice size was selected because the shear stress curves converge beyond this scale, indicating that larger systems do not significantly alter the results shown in Figure 7a–c.

**Figure 7:**
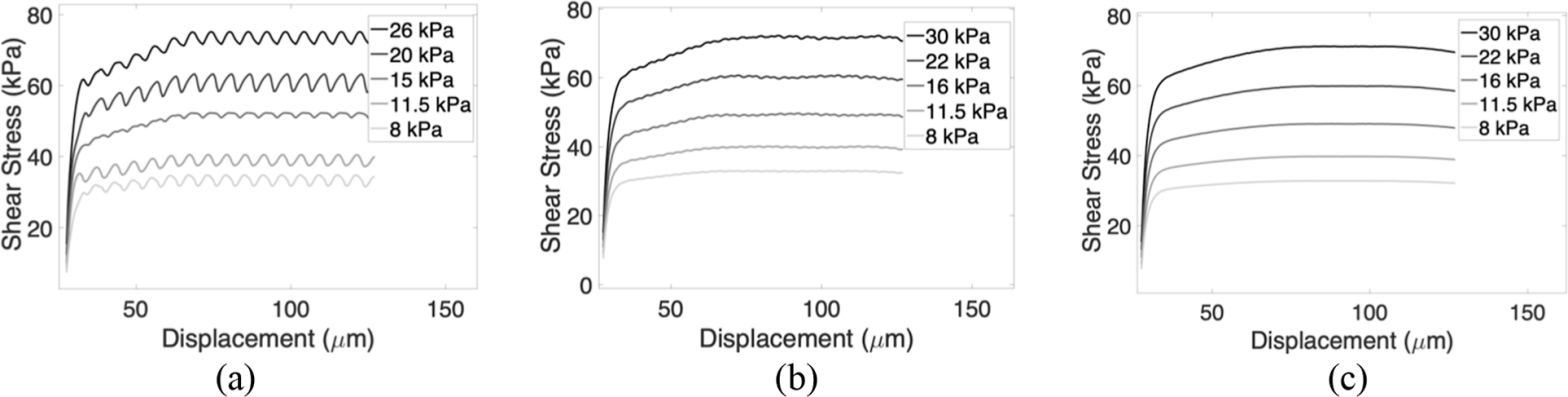
Friction simulation results for λ = 1.006 at various misorientation angles θ = 0°, 5°, and 15° and five different normal loads. (a) Shear stress vs shear displacement at θ = 0°. (b) Shear stress vs shear displacement at θ = 5°. (c) Shear stress vs shear displacement at θ = 15°.

Figure 7 presents the simulated shear stress versus displacement curves for several lattice misorientation angles. As shown in Figure 7a (0°), the shear stress exhibits a periodic oscillation arising from the registry between the two aligned lattices. However, this oscillatory response progressively diminishes with increasing misorientation: for 5° and 15° (Figure 7b,c), the curves become increasingly smooth. In all cases, the shear stress is computed by summing the lateral force on each top-layer pillar at every time step and dividing it by the total area of the top lattice.

We orient one of the lattices at a misorientation, θ, with respect to center to generate Moiré patterns or dislocations on the interface as shown in Figure 4a–c. Full simulation videos corresponding to cases in Figure 4a–c are shown in the Supporting Information (Section S6), namely: SV1, SV2, and SV3. The rest of the figures for cases of λ = 1, 1.023 are shown in Figures S5 and S7. Figure 4 shows the spatial maps of displacement between interacting pillars across top and bottom layers before sliding at various rotational offsets (0°, 5°, and 15°). Videos SV1, SV2, and SV3 show simulations corresponding to the three misorientation angles. When sliding begins, the lattice mismatch or an orientation mismatch gives rise to interfacial features resembling Moiré patterns. As the rotation angle increases, the pattern transitions from a uniform grid (0°) to Moiré-like interference structures (5°–45°) as also shown in Figure 3, reflecting how relative alignment, θ, affects the interface. As shown in Figures 3 and 4, the density of Moiré patterns increases with an increase in θ. Additional simulation videos for a smaller lattice size (∼200 pillars sliding over 700 pillars) for θ = 0° to 45° are available as Videos SV4, S5, S6, S7, S8, and S9 in the Supporting Information.

In our previous modeling framework,^22^ pillar interactions were treated as isolated pairwise contacts without accounting for the underlying substrate’s compliance. This simplification assumed that each pillar deformed independently, neglecting the mechanical coupling introduced by the shared substrate. As a result, shear stress versus displacement curves for θ = 0° exhibited a prominent sawtooth curve-like behavior, reflecting abrupt transitions in contact states between individual pillars. In contrast, the current simulation model incorporates substrate compliance, recognizing that all of the pillars are elastically coupled through a continuous substrate. This allows long-range stress redistribution and coordinated deformation across the pillar array. Consequently, for the same θ = 0° alignment case, the characteristic sawtooth profile observed previously transitions to a smoother force response as shown in Figure 7a, reflecting the collective influence of pillar and substrate-mediated interactions. Figure 7b,c refers to simulation results for θ = 5° and θ = 15° respectively. As shown, the shear stress becomes smoother as the misorientation increases, reflecting similar data from experiments. Note that the simulation (Figure 7) does not capture the static friction peak associated with the initiation of sliding (Figure 5a). The static friction peak is associated with different processes than sliding or dynamic friction; it often involves fracture or adhesion.^25^ Since our focus in this work is on sliding friction, we have not included adhesion in our models and consequently do not capture the static friction peak. We only show some of the cases here, and the rest of the cases of θ = 30°, 45°, and λ = 1, 1.023 for all θs are shown in Figures S6, S8, and S10.

Normal coupling between pillars is neglected based on the assumption that vertical compliance is localized beneath each pillar and that normal forces can be determined independently via local force balance. Since the model focuses on shear deformation with a one-way coupling to normal force, accounting for cross-pillar normal interactions is not required. In contrast, shear deformation involves a distributed substrate strain and must include coupling between pillars.

In a fully coupled model, both shear and normal displacements interact across and within layers. In our approach, we primarily model shear deformation and compute normal forces independently for each pillar through local force balance. While normal force coupling between pillars on the same layer is not explicitly included, it is indirectly captured because the normal force depends on the shear forces, which are coupled through the substrate.

We proceeded to calculate the normal force. Until this point, our analysis focused on shear forces and displacements in the *x*–*y* plane. In a full model, one would introduce additional kinematic variables such as “*z*” displacement and additional forces such as the “*z*” component of force. Here, we simplify the model to be in the *x*–*y* plane and provide one-way coupling via vertical force balance. In particular, this approach obviates the need to account for the z-displacement coupling between pillars.

We begin by examining a representative interaction between two pillars during sliding, as illustrated in Figure 8. Upon initial contact, the pillars start bending to accommodate the imposed sliding displacement. This bending persists until reaching a mechanical limit, beyond which the pillars can no longer sustain the deformation solely through bending. At this threshold, the pillars initiate sliding apart. During the bending process, both normal and shear forces arise concurrently. To understand their interplay, we employ a force balance approach that couples these two forces. As sliding initiates, assuming point contact, the pillars bend by an angle α, defined by the relationship: $\tan\alpha =\frac{\delta_{y}}{2l}$ , where δ_*y*_ represents the shear displacement and 2*L* denotes the effective contact height between the pillars. The shear force (*F*) is defined as the overall shear force observed on a single pillar in the *x* and *y* directions. Here, we assume Coulombic friction, and the total shear force *F* is resisted by the tangential component of the contact normal force (*C*_N_) and normal component of τ, where τ = μ*C*_N_, and μ is the coefficient of friction between pillars. The normal contact force (*C*_N_) decomposes into components as *C*_N_cos α and *C*_N_sin α in horizontal and vertical directions, respectively. τ also decomposes into τ sin θ and τ cos θ. The shear force can be written as *F* = *C*_N_ cos α + μ*C*_N_ sin α. This gives, *F* = *C*_N_(cos α + μ sin α), and thus, $C_{N}=\frac{F}{\cos\;\alpha +\mu \;\sin\alpha }$ . The total normal force on a single pillar (*N*) can be defined as a function of shear force. From the force balance, we get normal force as *N* = *C*_N_(sin α – μ cos α). Substituting *C*_N_ to get normal force from given shear force as

19 $$N=\frac{F}{\left(\cos\;\alpha +\mu \times \sin\alpha \right)}\times \left(\sin\alpha -\mu \times \cos\;\alpha \right)$$

**Figure 8:**
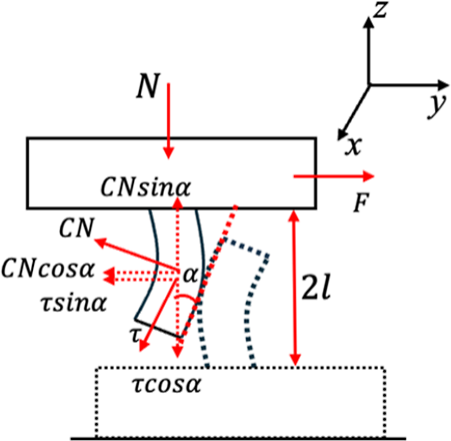
Free body diagram illustrating the interaction of two contacting pillars undergoing lateral sliding. Upon contact, bending induces both a shear force and a normal force. The angle of bending, α, reflects the geometry via $\tan\alpha =\frac{\delta_{y}}{2l}$ , where δ_*y*_ is the shear displacement and 2*L* is the effective contact height.

This final expression clearly illustrates how the normal force acting on pillars systematically depends on the known shear force measurements, geometry (α, δ_*y*_, *l*), and frictional parameter (μ). It underscores the interdependence of normal and shear forces.

Figure 9a,b shows a snapshot of the interface during sliding friction experiment and simulation at θ = 5°. Figure 9a corresponds to Moiré patterns as seen in micropillar experiments, and Figure 9b corresponds to Moiré patterns as they appear in our layer-based model simulation. The color scale represents the amount of relative shear displacement between pillar pairs: black indicates maximum displacement in sliding direction, while lighter shades of red indicate lesser relative displacement between the two interacting pillars. Sliding friction simulation is performed for 1700 pillars sliding over a lattice of 3200 pillars. The normal stress is computed as the sum of normal loads on all pillars on the upper part, divided by the interfacial area. Figure 9c shows the friction stress versus normal stress from simulations at θ = 0° to 45° at λ = 1.006 in comparison to stress data from the experiments. As shown in Figure 9c, our model results compare well with those of our experiments.

**Figure 9:**
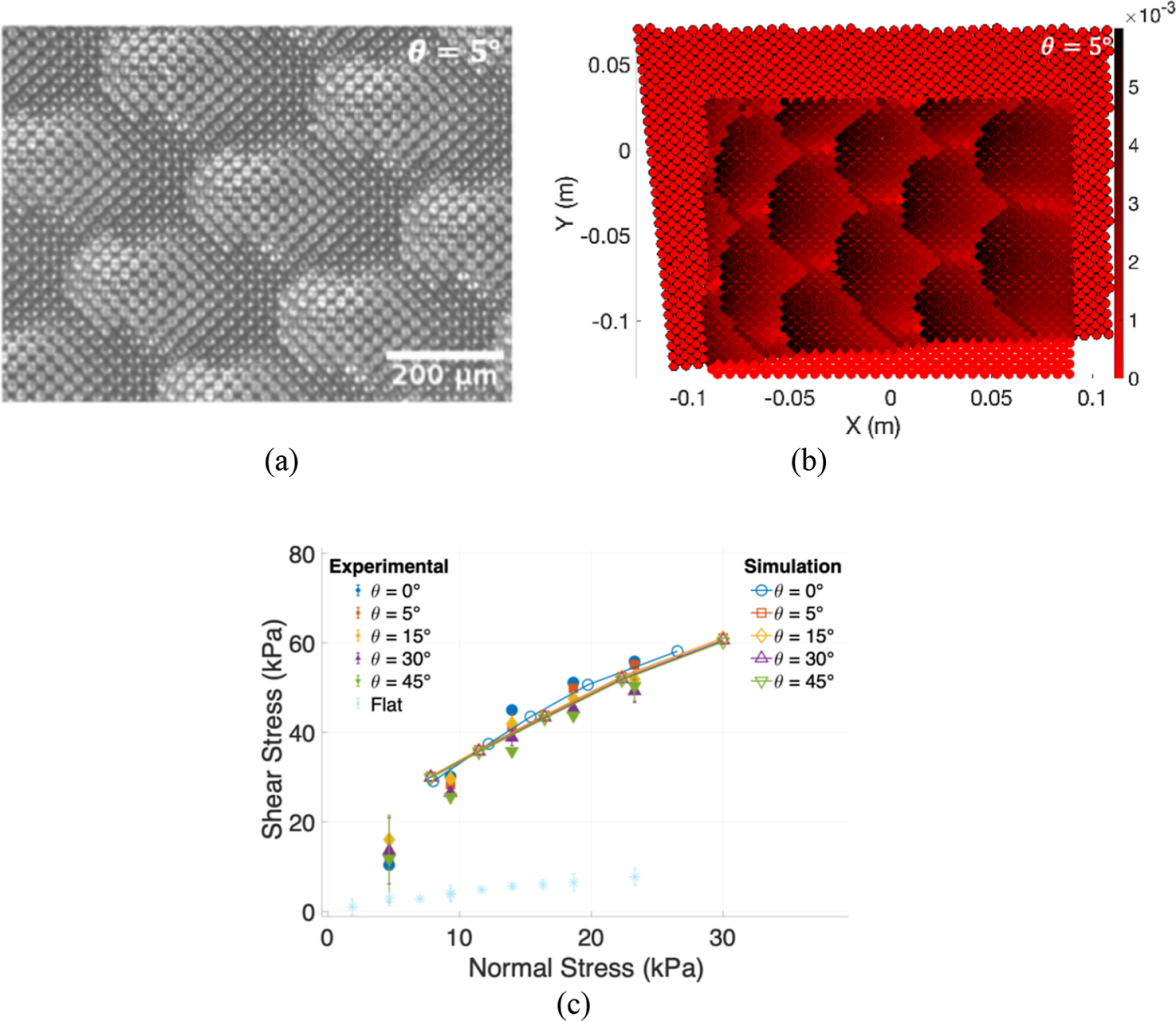
Comparison of experimental and simulated images of the interface and friction stress for varying normal stress. (a) Interfacial dislocations as seen in experiments during sliding. (b) Simulated sliding of a lattice containing 1700 pillars over 3200 pillars. The color bar refers to the local value of pillar deflection. (c) Friction stress vs normal stress from simulation in comparison to our stress data from the experiments.

### 3.5 FEA Simulation of Pillar Layer Sliding

We perform FE analysis of pillar sliding to capture the overall sliding and forces and to validate the layer-based model using ABAQUS 2023 (Dassault Systèmes, Providence, RI, USA). As shown in Figure 10, the model consists of two parts: a bottom part containing 25 pillars (yellow) arranged in a square array with a center-to-center spacing of *a* = 4*R* = 6 mm along two orthogonal in-plane directions and a top part containing a single pillar (green) located at the center. All pillars have a diameter of *D* = 2*R* = 3 mm and a height of *h* = 4.8 mm. The bottom part includes a substrate measuring 30 × 30 mm, while the substrate of the top part is smaller, 12 × 12 mm, to reduce computational cost. Both parts have a uniform thickness of *T* = 12 mm in the vertical direction. Figure 10a shows the top view of the model geometry and Figure 10b shows the front view. Interaction between the top and bottom parts is defined using a friction coefficient of 0.4. To prevent interpenetration between pillars on the same part and between pillars and the substrate surface, we apply hard contact.

**Figure 10:**
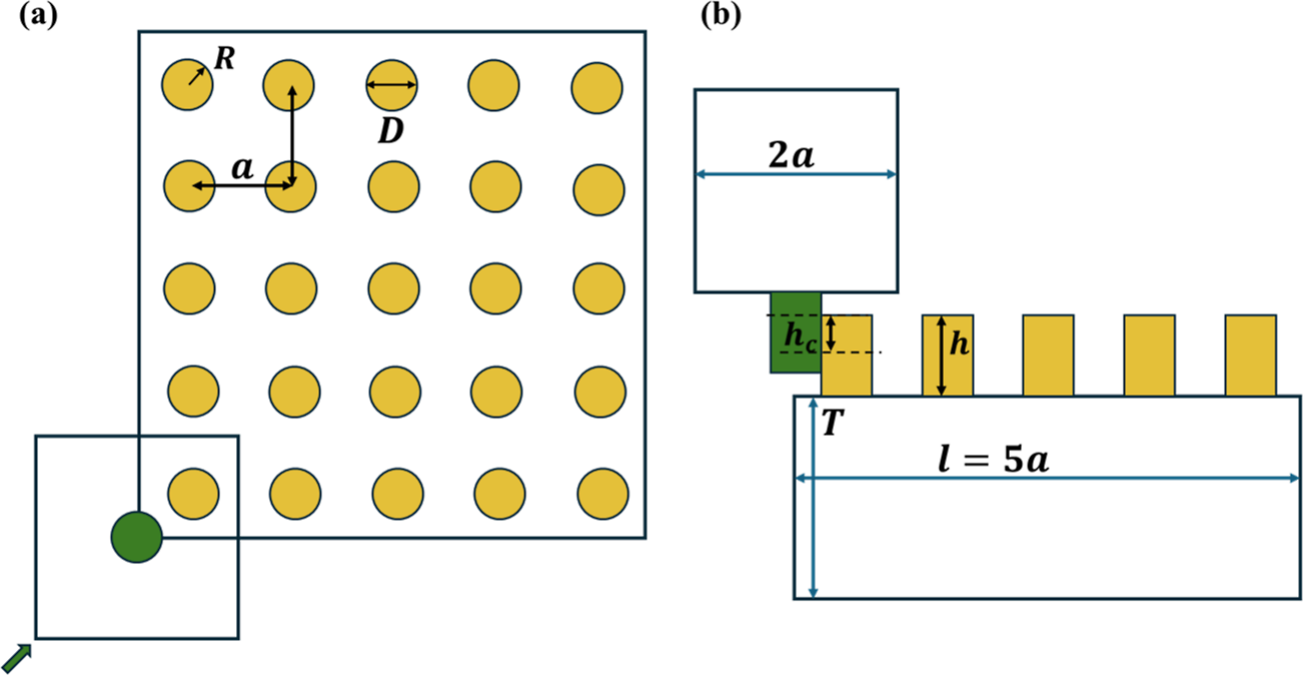
Geometry of the simulation model. (a) Top view and (b) front view. The model consists of a top part with one central pillar (green) and a bottom part with 25 periodically arranged pillars (yellow). All pillars have diameter *D* = 3 mm and spacing *a* = 6 mm. Both substrates have a thickness of *T* = 12 mm. The bottom part is fixed at its bottom; the top part moves diagonally while being constrained in height.

At the start of the simulation, the center of the top part is positioned at the edge of the bottom part with a height overlap between the two sets of pillars defined as *h*_c_ (Figure 10b). The pillars are initially separated and not in contact. A displacement-controlled motion is applied to the top surface of the top part, moving it diagonally in the plane at a velocity of $\sqrt{2}/2\times 10^{-3}\;m/s$ , while keeping its height fixed. The bottom part is fully fixed at its bottom surface. Both parts are modeled as incompressible neo-Hookean materials, with a shear modulus of μ_0_ = 1 MPa and a mass density of ρ = 965 kg/m^3^.^26^ The simulations are performed using the dynamic implicit, quasi-static solver in ABAQUS, meaning that inertial effects are neglected; the density is included primarily to regularize the problem and to enable the simulation of unstable events, which cannot be captured using a purely static solver. Incompressibility is enforced by setting bulk modulus *K* = 2000 MPa. Following our previous work and to match the experimental results, the shear modulus is scaled to μ = 0.65 MPa. Accordingly, the forces used for comparison with the layer-based model results are obtained by multiplying the original FE output by a scaling factor of 0.65.

All elements are of type C3D8RH. The mesh is shown in Figure 11, where Figure 11a presents the mesh for the bottom part and Figure 11b presents the mesh for the top part. Note that the scale is not consistent between the two views; Figure 11b is enlarged to show mesh details. For *h*_c_ = 2.4 mm and *h*_c_ = 3.6 mm, the mesh size on the pillars (top and bottom parts) is uniformly 0.2 mm along both radial and axial directions. In the in-plane substrate regions, the mesh size increases from 0.2 mm at the pillar edge to 0.8 mm at the substrate edge for the top part and to 0.4 mm at the substrate edge for the bottom part. For the top part, we added an additional square partition centered on the part, with a side height of 4*R* and applied a mesh size of 0.4 mm on this square to facilitate a smoother transition from the fine mesh near the pillar to the coarser mesh toward the edge of the substrate. Along the thickness direction of the substrates, the mesh is refined to 0.2 mm within the first 0.6 mm from the interface with pillars, then gradually coarsens to 0.8 mm over the next 1.8 mm, and remains at 0.8 mm through the remainder of the domain. For the case of *h*_c_ = 1.2 mm, a finer mesh is used for the single pillar in the top part: 0.1 mm along the axial direction and radially refined from 0.2 mm at the center to 0.1 mm near the edge. All other mesh settings remain the same as in the *h*_c_ = 2.4 mm and *h*_c_ = 3.6 mm cases. A mesh convergence test was performed by using a smaller system consisting of only two diagonally positioned pillars on the bottom part (with a correspondingly smaller substrate size). Further details are provided in the Supporting Information, Figures S17–S19.

**Figure 11:**
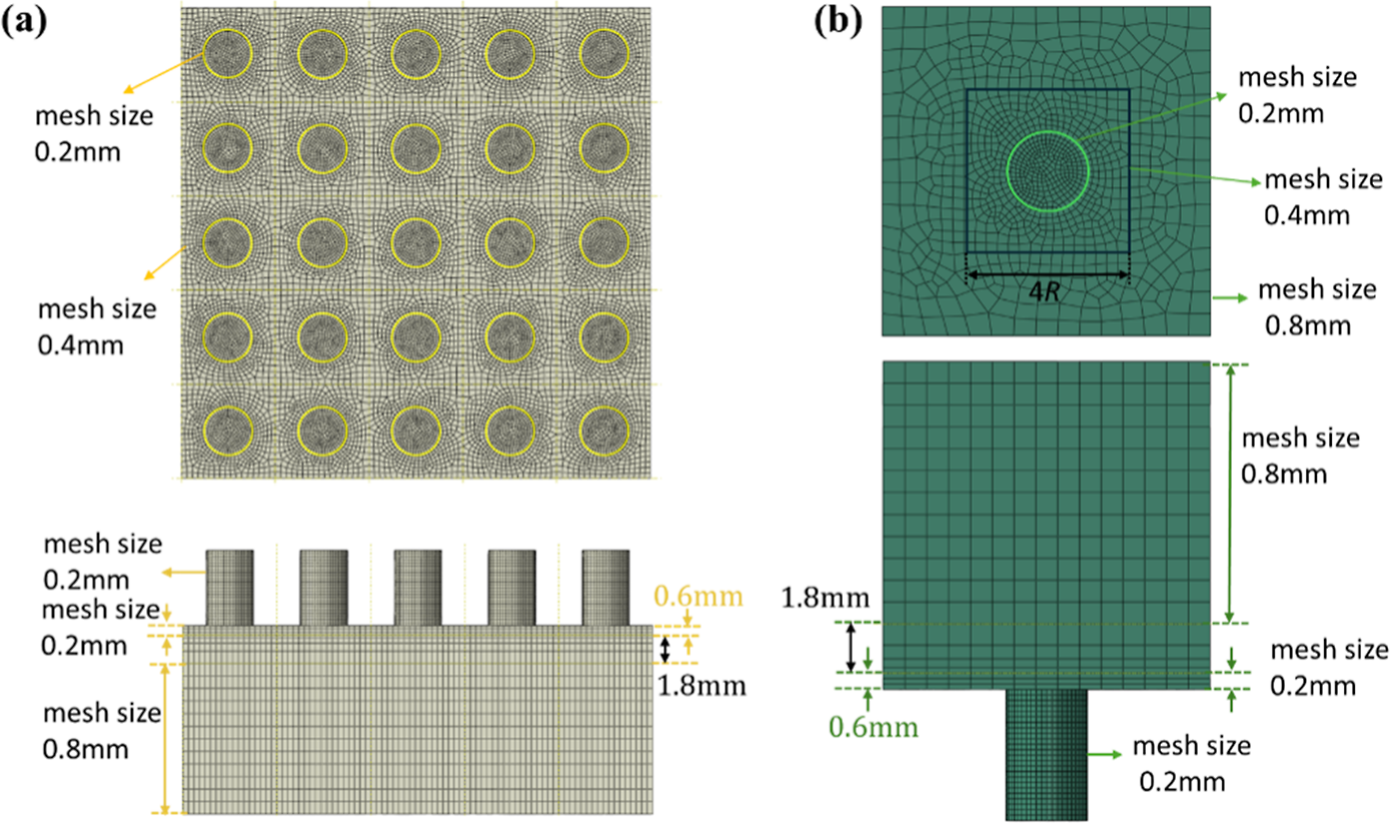
FE mesh of the simulation model. (a) Mesh of the bottom part with 25 pillars; edges of pillars are highlighted in yellow circles. (b) Mesh of the top part with one central pillar highlighted in green. Note that the scale differs between (a,b); the top part is enlarged to show mesh details.

The simulation results of shear and normal reaction forces applied to the top surface of the top part are shown in Figure 12a,b, respectively, as a comparison with results from our layer-based simulation for a pillar sliding over the 5 × 5 matrix of bottom pillars. Each plot includes data for three cases: *h*_c_ = 1.2 mm, *h*_c_ = 2.4 mm, and *h*_c_ = 3.6 mm. All reaction forces are scaled by a factor of 0.65 to account for the adjusted shear modulus. The validation linear scaling is confirmed in Figure S19. Spikes appear in the force curves when the top pillar transitions from contact with one bottom pillar to the next, likely due to the high contact stiffness introduced by the penalty method. The raw simulation data, including these spikes, is provided in Figures S13–S15 of the Supporting Information. The strong agreement in normal force results validates the reliability of our layer-based model in capturing the dependence of normal force on shear deformation, supporting its use in predictive studies.

**Figure 12:**
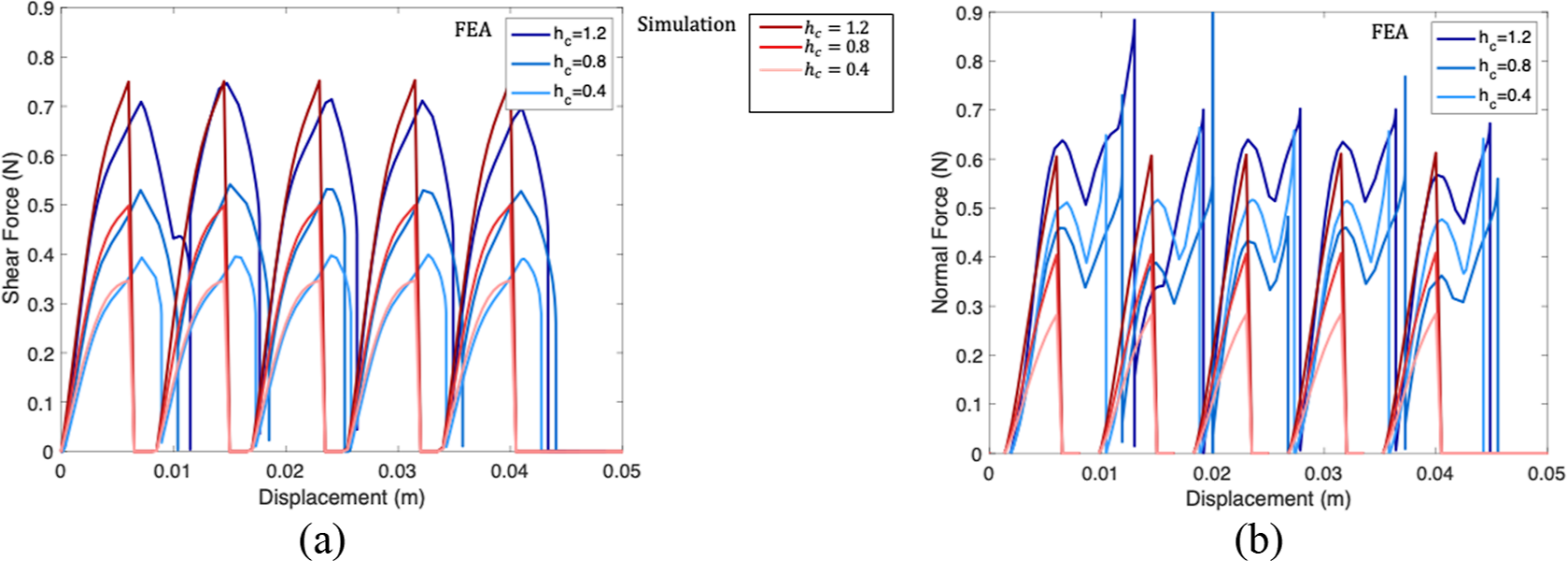
Comparison of results from FEA and the layer-based model. (a) Shear force vs shear displacement for a configuration with one top pillar and 25 bottom pillars, shown for three different contact heights. (b) Normal force vs shear displacement for the same configuration and contact height conditions.

## 4 Summary and Conclusion

This study focuses on understanding sliding friction in microscale pillar arrays using a layer-based model. This model simulates interactions between top and bottom pillar arrays by calculating forces based on their proximity. The model captures the origin of shear friction as arising from both pillar–pillar interactions at the sliding interface and elastic coupling between pillars within the substrate. The system is represented as four layers of nodes, where displacement is applied to the top layer and deformation propagates through substrate and beam-like interactions across the interface. By accounting for misorientation and height overlap between pillars, the model successfully predicts friction forces and deformation patterns that align well with experimental observations from multifiber sliding tests. Our model successfully captures frictional forces for an array of sliding pillars and helps us visualize the Moiré patterns while sliding.

## Supplementary Material







































## Notes

Sliding of 30×30 (∼1770) pillars on 40×40 (∼3160) array at 0° orientation, θ and λ = 1.006 (MP4)
Sliding of 30×30 (∼1770) pillars on 40×40 (∼3160) array at 5° orientation, θ and λ = 1.006, in layer-based simulation (MP4)
Sliding of 30×30 (∼1770) pillars on 40×40 (∼3160) array at 15° orientation, θ and λ = 1.006, in layer-based simulation (MP4)
Sliding of 10×10 (∼190) pillars on 20×20 (∼728) array at 0° orientation at stiffness 2 MPa, in layer-based simulation (MP4)
Sliding of 10×10 (∼190) pillars on 20×20 (∼728) array at 0° orientation at stiffness 200 kPa, in layer-based simulation (MP4)
Sliding of 10×10 (∼190) pillars on 20×20 (∼728) array at 5° orientation at stiffness 2 MPa, in layer-based simulation (MP4)
Sliding of 10×10 (∼190) pillars on 20×20 (∼728) array at 15° orientation at stiffness 2 MPa, in layer-based simulation (MP4)
Sliding of 10×10 (∼190) pillars on 20×20 (∼728) array at 30° orientation at stiffness 2 MPa, in layer-based simulation (MP4)
Sliding of 10×10 (∼190) pillars on 20×20 (∼728) array at 45° orientation at stiffness 2 MPa, in layer-based simulation (MP4)
Sliding of 10×10 (∼190) pillars on 20×20 (∼728) array at 0° orientation at stiffness 2 MPa, in layer-based simulation. Recording with the corresponding force plot (MP4)
Sliding of 10×10 (∼190) pillars on 20×20 (∼728) array at 0° orientation at stiffness 200 kPa, in layer-based simulation (MP4)
Sliding friction experiment at θ = 0°, 5x magnification, −1 V normal load, λ = 1.006 (MP4)
Sliding friction experiment at θ = 5°, 5x magnification, −0.5 V normal load, λ = 1.006 (MP4)
Sliding friction experiment at θ = 15°, 5x magnification, −0.5 V normal load, λ = 1.006 (MP4)
Sliding friction experiment at θ = 30°, 5x magnification, −0.5 V normal load, λ = 1.006 (MP4)
Sliding friction experiment at θ = 45°, 5x magnification, −0.5 V normal load, λ = 1.006 (MP4)
Sliding friction experiment of pillar-pillar interface at θ = 0°, 5x magnification, −0.5 V normal load, λ = 1 (MP4)
Sliding friction experiment of pillar-pillar interface at θ = 5°, 5x magnification, −0.5 V normal load, λ = 1 (MP4)
Pillar friction data (λ = 1); pillar friction data (λ = 1.023); derivation of stiffness coefficients from contact mechanics; photos and videos from simulation; and FE simulation using ABAQUS S5 (PDF)
