# Supplementary material for: A Layer-Based Model for Frictional Sliding of Pillar Arrays
Source: Langmuir. 2026 Feb 10;42(7):5440–51. doi: 10.1021/acs.langmuir.5c05261 (PMC12937109; doi:10.1021/acs.langmuir.5c05261)
Supplement: Supplementary file 19 [file la5c05261_si_019.pdf]

Supplementary Information

A Layer-Based Model for Frictional Sliding of Pillar Arrays

Jasreen Kaur[1], Xuemei Xiao[3], Preetika Karnal[1], Chung Yuen Hui[3], Anand Jagota[1,2*]

[1]Department of Chemical & Biomolecular Engineering, Lehigh University, Bethlehem, PA 18015 (USA)

[2]Department of Bioengineering, Lehigh University, Bethlehem, PA 18015 (USA)

[3]Cornell University, Ithaca, New York 14853 (USA)

* Corresponding author, email: anj6@lehigh.edu

S1.1 Pillar friction data ($\lambda = 1$)

Figure S1 shows interfacial dislocations between micropillar samples at $\lambda = 1$ for a range of misorientations θ from 0° to 45°. At $\theta = 0°$, the interface is fully aligned with no dislocations present. As the misorientation increases to 5°, 15°, 30°, and 45°, interfacial screw dislocations appear and become progressively denser. These images illustrate how misorientation induces dislocation structures at the interface, with their density and arrangement varying systematically with angle. Videos for the case of $\lambda = 1$ at $\theta = 0°$ and 5° are listed below as SV.17 and SV.18 (section S5) and videos at all misorientations for the case of $\lambda = 1.006$ are listed from SV.12 to SV.16 (section S5).

Figure S2 shows shear stress vs displacement experimental results for $\lambda = 1$ at several normal loads ranging from 4 kN/m^2 to 23 kN/m^2. Figure S2 (a) shows experimental results for shear stress versus displacement

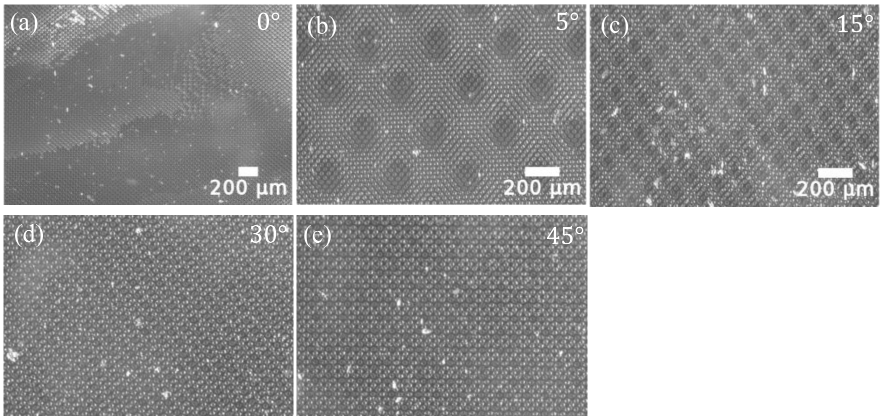

Figure S1. Interfacial screw dislocations
Interfacial dislocations between micropillar samples at $\lambda = 1$ for different misorientations θ. a) $\theta = 0°$ $b)$ $\theta = 5°$ $c)$ $\theta = 15°$ $d)$ $\theta = 30°$ $e)$ $\theta = 45°$.

for $\theta = 0°$ (b) $\theta = 5°$ (c) $\theta = 15°$ (d) $\theta = 30°$ (e) $\theta = 45°$. Figure S2 (f) shows shear stress versus normal stress for $\lambda = 1$ in comparison with a control sample.

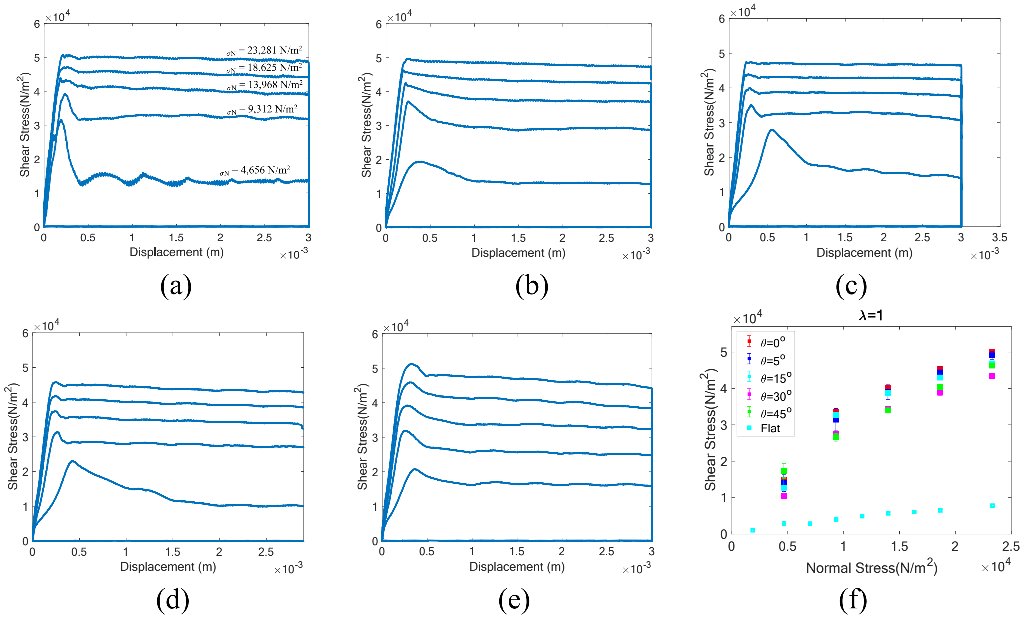

Figure S2. $\boldsymbol{\lambda = 1}$: This figure represents friction data for $\lambda = 1$ at various misorientations $\theta = 0°, 5°, 15°, 30°$ & $\theta = 45°$ and normal loads from 4 kN/m^2 to 23 kN/m^2. (a) Friction stress vs shear displacement for $\theta = 0°$ for varying normal load. (b) $\theta = 5°$ (c) $\theta = 15°$ (d) $\theta = 30°$ (e) $\theta = 45°$ (f) Friction stress vs normal stress for $\lambda = 1$ and at various misorientations $\theta = 0°, 5°, 15°, 30°$ & $\theta = 45°$ and normal load varying from 4 kN/m^2 to 23 kN/m^2 in comparison to flat control.

S1.2 Pillar friction data ($\lambda = 1.023$)

The figure S3 below shows the interfacial dislocations for $\lambda = 1.023$ at several misorientations ranging from 0° to 45°. Density of dislocations depends on orientation as shown in the figures and also in previous research [1] where, ρ (linear density) can be understood to define the normalized spacing between dislocation features across two misoriented structures, where λ is scaling factor between two structures and θ is misorientation angle between them. The second quantity, α, gives the orientation of the Moire pattern/dislocation and can be geometrically interpreted as the angle between two vectors [1].

$$\rho = \sqrt{1 + \lambda^2 - 2\lambda os\theta} \quad \text{(S1)}$$

$$\alpha = tan^{-1}\left(\frac{sin\theta}{\lambda - cos\theta}\right) \quad \text{(S2)}$$

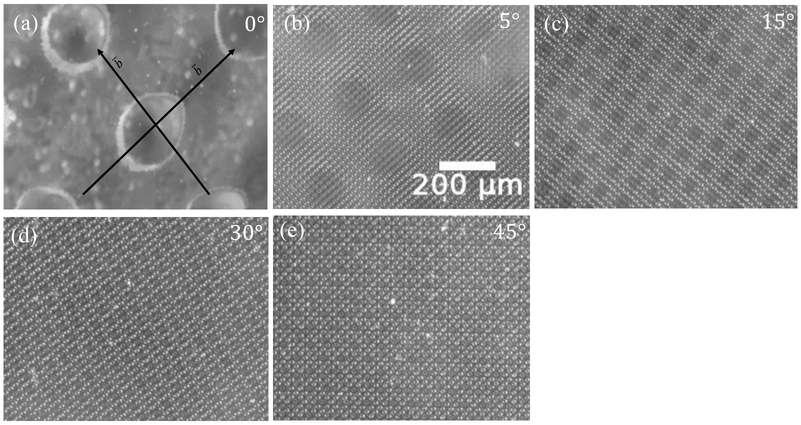

Figure S3. $\lambda = 1.023$ Interfacial dislocations between micropillar samples at $\lambda = 1.023$ for different misorientations between the top and bottom samples. $a)\ \theta = 0°\ b)\ \theta = 5°\ c)\ \theta = 15°\ d)\ \theta = 30°\ (e)\ \theta = 45°$.

Figure S4 shows experimental results for $\lambda = 1.023$ at several normal loads ranging from 4000 N/m^2 to 23000 N/m^2. Figure S4 (a) shows experimental results for shear stress versus displacement for $\theta = 0°$ (b) $\theta = 5°$ (c) $\theta = 15°$ (d) $\theta = 30°$ (e) $\theta = 45°$. Figure S4 (f) shows the variation of shear stress with normal stress for $\lambda = 1.023$ and different misorientations between the top and bottom samples.

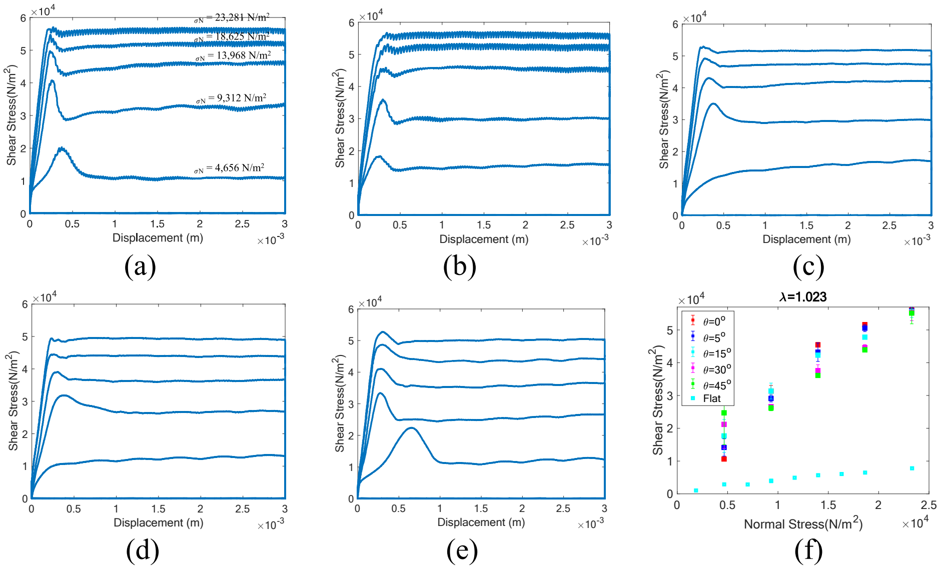

Figure S4. $\lambda = 1.023$: This figure represents friction data for $\lambda = 1.023$ at various misorientations $\theta = 0°, 5°, 15°, 30°\ \&\ \theta = 45°$. (a) Friction stress vs shear displacement for $\theta = 0°$ for normal load varying from 4000 N/m^2 to 23000 N/m^2 (b) $\theta = 5°$ (c) $\theta = 15°$ (d) $\theta = 30°$ (e) $\theta = 45°$ (f) Friction stress vs normal stress for $\lambda = 1.023$ and at various misorientations $\theta = 0°, 5°, 15°, 30°\ \&\ 45°$ and normal load varying from 4000 N/m^2 to 23000 N/m^2.

S2. Derivation of stiffness coefficients from contact mechanics.

The substrate stiffness coefficients used in the main section to define interaction of pillars can be derived by making appropriate assumptions about the traction distribution on the interface between the pillars and substrate and using results from Contact Mechanics [2]. Consider the tangential surface displacement, Δu_x, that arises from applying a *specific* spatially varying tangential traction over a circular contact region of radius a, given by

$$q_x(x,y) = q_o\sqrt{1-\frac{r^2}{a^2}} \tag{S3}$$

Although the traction is non-uniform, maximized at the center and vanishing at the boundary, this distribution is chosen such that the resulting surface displacement within the contact area is uniform. The displacement Δu_x, is independent of position within the circle and is given by the closed-form expression (Equation 3.86a from [2])

$$\Delta u_x = \frac{q_o a\pi(2-\nu)}{4G} \tag{S4}$$

where ν is Poisson's ratio and G is the shear modulus of the elastic half-space. This result is valid for arbitrary values of ν, not just for the incompressible case, and demonstrates that an appropriately selected tangential loading can produce a constant displacement field despite spatial variation in applied stress. q_o can be written as $q_o = \frac{Q_x}{4\pi a^2}$, and thus the stiffness coefficient at a point $r < a$, can be written as

$$\Delta u_x = \frac{Q_x}{4\pi a^2}.\frac{\pi a(2-\nu)}{4G}, k_s = \frac{16Ga}{2-\nu} \tag{S5}$$

This gives, k_s, where G is the shear modulus, ν is Poisson's ratio.

Moving on to a point outside a, we consider displacements resulting from tangential point force, Q_x. A concentrated tangential force Q_x is applied at the origin of an elastic half-space parallel to the x-axis. The

normal pressure, $p = 0$, and there is no tangential traction in the y-direction, $q_y = 0$. Assuming $q_x(x,y)$ is concentrated at a single point: the origin, the total force is

$$Q_x = \iint q_x(x,y)dxdy \tag{S6}$$

Thus, traction becomes a point force, and expressions for displacement at any point (x,y,z) in the half-space are (Equation 3.75 a, b, c from [2])

$$\Delta u_x = \frac{Q_x}{4\pi G}\left[\frac{1}{\rho} + \frac{x^2}{\rho^3} + (1-2\nu)\left\{\frac{1}{\rho+z} - \frac{x^2}{\rho(\rho+z)^2}\right\}\right] \tag{S7}$$

$$\Delta u_y = \frac{Q_x}{4\pi G}\left[\frac{xy}{\rho^3} - (1-2\nu)\left\{\frac{xy}{\rho(\rho+z)^2}\right\}\right] \tag{S8}$$

$$\Delta u_z = \frac{Q_x}{4\pi G}\left[\frac{xz}{\rho^3} + (1-2\nu)\left\{\frac{x}{\rho(\rho+z)}\right\}\right] \tag{S9}$$

where, $\rho^2 = x^2 + y^2 + z^2$

Since for our case, $\nu = 0.5$, the second half of these equations go to zero. Considering the stress and displacement are on the surface, thus, $z = 0$, $\rho = r$. Thus,

$$\Delta u_x = \frac{Q_x}{4\pi G}\left[\frac{1}{\rho} + \frac{x^2}{\rho^3}\right] \tag{S10}$$

$$\Delta u_y = \frac{Q_x}{4\pi G}\left[\frac{xy}{\rho^3}\right] \tag{S11}$$

$$u_z = 0 \tag{S12}$$

From above equations stiffness coefficients for concentrated tangential force on pillar *i* and its effect on pillar *j* can be written as

$$k_{sijx} = 4\pi G\left[\frac{1}{\rho} + \frac{x^2}{\rho^3}\right]^{-1} \tag{S13}$$

$$k_{sijy} = 4\pi G\left[\frac{1}{\rho} + \frac{y^2}{\rho^3}\right]^{-1} \tag{S14}$$

$$k_{sijxy} = 4\pi G\left[\frac{xy}{\rho^3}\right]^{-1} \tag{S15}$$

Moving to the expressions of Δu_x and Δu_y for the case where on pillar *i* is applied a tangential force, F_{six} which leads to a displacement in *x* direction. If there is no elastic coupling between the pillars on the substrate, the displacement at *i* is,

$$\Delta u_{ix} = \frac{F_{six}}{k_s} \tag{S16}$$

When there is elastic coupling involved, a tangential force F_{six} on pillar *i* causes a displacement on *i,* and on neighboring pillar *j,* such that the displacement on pillar *j* in *x* and *y* directions can be written as

$$\Delta u_{x,ij} = \frac{F_{six}}{4\pi G}\left[\frac{1}{\rho} + \frac{x^2}{\rho^3}\right] \tag{S17}$$

$$\Delta u_{yij} = \frac{F_{six}}{4\pi G}\left[\frac{xy}{\rho^3}\right] \tag{S18}$$

(this is due to force on *i* and displacement on *j*). The displacement on *j* also creates a force F_{sjy} on *j* which then leads to an effective displacement on *i,* written as

$$\Delta u_{yji} = \frac{F_{sjy}}{4\pi G}\left[\frac{1}{\rho} + \frac{y^2}{\rho^3}\right] \tag{S19}$$

$$\Delta u_{xji} = \frac{F_{sjy}}{4\pi G}\left[\frac{xy}{\rho^3}\right] \tag{S20}$$

Thus, the overall displacement on *i* (due to force on itself and all other pillars j on the same layer) in *x* and *y* directions becomes,

$$\Delta u_{ix} = \frac{F_{six}}{k_s} + \sum_{j=1}^{N} \frac{F_{sjx}}{k_{sijx}} + \frac{F_{sjy}}{k_{sijxy}} \tag{S21}$$

$$\Delta u_{iy} = \frac{F_{siy}}{k_s} + \sum_{j=1}^{N} \frac{F_{sjy}}{k_{sijy}} + \frac{F_{sjx}}{k_{sijxy}} \tag{S22}$$

Each pillar experiences a displacement not only from its own restoring force (through k_s), but also from distant forces applied to other pillars. These remote contributions decay as $1/\rho$ or faster, reflecting the elastic nature of the substrate.

These expressions (S21 and S22) can be compactly written in matrix form to describe the displacement of all pillars in the system. Letting Δu be the vector of displacements and F_s the vector of substrate forces, we write:

$$\{\Delta u\} = [C]_s\{F_s\},\ \{F_s\} = [C]_s{}^{-1}\{\Delta u\} \tag{S23}$$

$[C]_s$ is the substrate compliance matrix, consisting of self-compliances (diagonal) and interaction compliances (off-diagonal).

$$\begin{Bmatrix} \Delta u_{1x} \\ \Delta u_{2x} \\ \cdots \\ \Delta u_{Nx} \\ \Delta u_{1y} \\ \Delta u_{2y} \\ \cdots \\ \Delta u_{Ny} \end{Bmatrix} = [C]_f \begin{Bmatrix} F_{s1x} \\ F_{s2x} \\ \cdots \\ F_{sNx} \\ F_{s1y} \\ F_{s2y} \\ \cdots \\ F_{sNy} \end{Bmatrix}; \begin{Bmatrix} F_{s1x} \\ F_{s2x} \\ \cdots \\ F_{sNx} \\ F_{s1y} \\ F_{s2y} \\ \cdots \\ F_{sNy} \end{Bmatrix} = \{F_f\} = [K]_f \begin{Bmatrix} \Delta u_{1x} \\ \Delta u_{2x} \\ \cdots \\ \Delta u_{Nx} \\ \Delta u_{1y} \\ \Delta u_{2y} \\ \cdots \\ \Delta u_{Ny} \end{Bmatrix}; [C]_f = [K]^{-1}{}_f \quad \text{(S24)}$$

where compliance matrix can be defined as

$$C = \begin{bmatrix} \frac{1}{k_s} & \frac{1}{k_{sx(1,2)}} & \frac{1}{k_{sx(1,3)}} & \cdots & \frac{1}{k_{sx(1,n)}} & \frac{1}{k_{sxy(1,1)}} & \frac{1}{k_{sxy(1,2)}} & \frac{1}{k_{sxy(1,3)}} & \cdots & \frac{1}{k_{sxy(1,n)}} \\ \frac{1}{k_{sx(2,1)}} & \frac{1}{k_s} & \frac{1}{k_{sx(2,3)}} & \cdots & \frac{1}{k_{sx(2,n)}} & \frac{1}{k_{sxy(2,1)}} & \frac{1}{k_{sxy(2,2)}} & \frac{1}{k_{sxy(2,3)}} & \cdots & \frac{1}{k_{sxy(2,n)}} \\ \frac{1}{k_{sx(3,1)}} & \frac{1}{k_{sx(3,2)}} & \frac{1}{k_s} & \cdots & \frac{1}{k_{sx(3,n)}} & \frac{1}{k_{sxy(3,1)}} & \frac{1}{k_{sxy(3,2)}} & \frac{1}{k_{sxy(3,3)}} & \cdots & \frac{1}{k_{sxy(3,n)}} \\ & & & & \vdots & & & & & \\ \frac{1}{k_{sx(n,1)}} & \frac{1}{k_{sx(n,2)}} & \frac{1}{k_{sx(n,3)}} & \cdots & \frac{1}{k_{sx(n,n)}} & \frac{1}{k_{sxy(n,1)}} & \frac{1}{k_{sxy(n,2)}} & \frac{1}{k_{sxy(n,3)}} & \cdots & \frac{1}{k_{sxy(n,n)}} \\ \frac{1}{k_{sxy(1,1)}} & \frac{1}{k_{sxy(1,2)}} & \frac{1}{k_{sxy(1,3)}} & \cdots & \frac{1}{k_{sxy(1,n)}} & \frac{1}{k_s} & \frac{1}{k_{sy(1,2)}} & \frac{1}{k_{sy(1,3)}} & \cdots & \frac{1}{k_{sy(1,n)}} \\ & & & & \vdots & & & & & \\ \frac{1}{k_{sxy(n,1)}} & \frac{1}{k_{sxy(n,2)}} & \frac{1}{k_{sxy(n,3)}} & \cdots & \frac{1}{k_{sxy(n,n)}} & \frac{1}{k_{sy(n,1)}} & \frac{1}{k_{sy(n,2)}} & \frac{1}{k_{sy(n,3)}} & \cdots & \frac{1}{k_s} \end{bmatrix}$$

S3 Photos and videos from simulation

S3.1 Simulation for ($\lambda = 1$)

The figure S5 presents simulation results showing interfacial dislocation patterns between micropillar samples with a periodicity ratio $\lambda = 1$ at different misorientation angles, θ. Figures S5 (a)–(e) correspond to increasing misorientation: $\theta = 0°, \theta = 5°,\ \theta = 15°,\ \theta = 30°, \theta = 45°$. At $\theta = 0°$, the micropillars are fully aligned, and no dislocation structures are observed. The color scale represents the amount of relative shear displacement between pillar pairs: black indicates maximum displacement in sliding direction**,** while lighter shades of red indicate less relative displacement between the two interacting pillars. As θ increases, interfacial screw dislocations emerge and progressively densify, forming dense patterns at higher angles.

These simulation results quantitatively and qualitatively match the trends observed in experiments, confirming that misorientation plays a critical role in generating interfacial dislocations between structured surfaces. The agreement between simulation and experimental data supports the validity of the modeled interaction rules and provides a robust framework for interpreting interfacial mechanics in pillar systems.

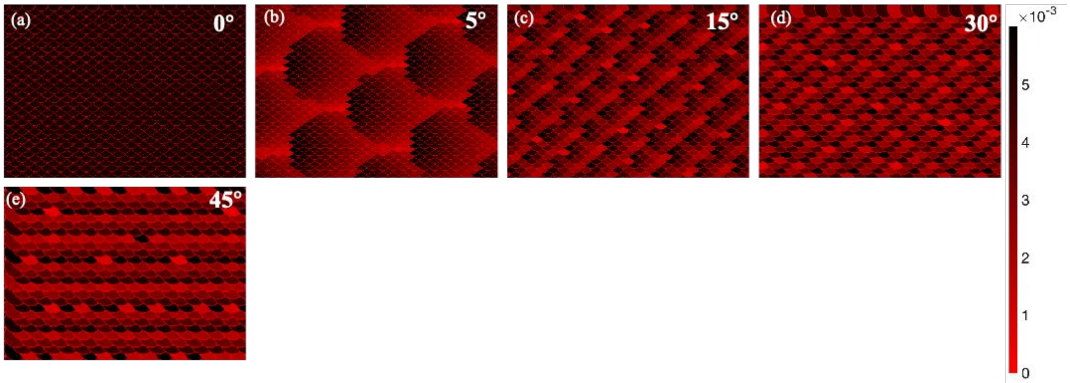

Figure S5. Simulation results: Interfacial dislocations between micropillar samples at $\lambda = 1$ at different misorientations. $a)\ \theta = 0°\ b)\ \theta = 5°\ c)\ \theta = 15°\ d)\ \theta = 30°\ (e)\ \theta = 45°$.

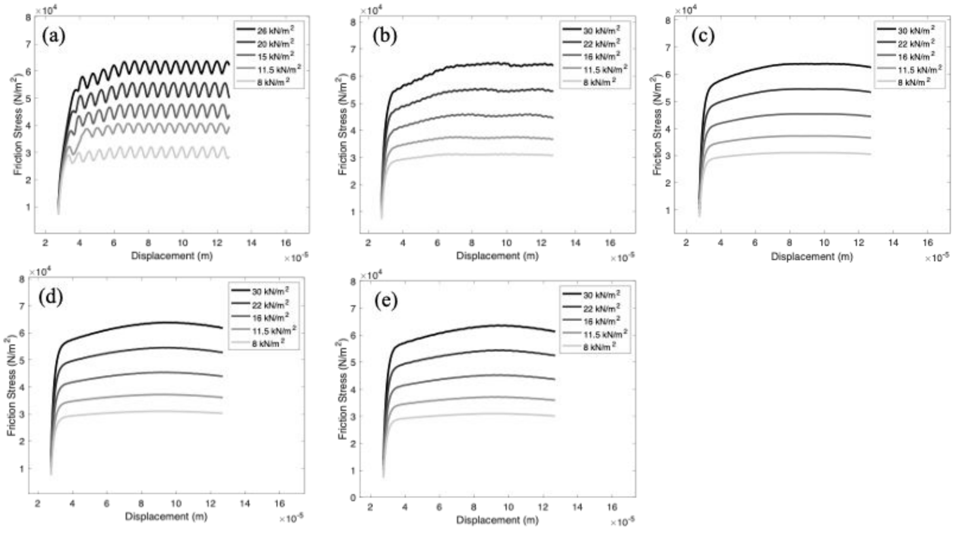

Figure S6 This figure represents friction data for $\lambda = 1$ at various misorientations $\theta = 0°, 5°, 15°, 30°\ \&\ \theta = 45°$ and five different normal loads. (a) Friction stress vs shear displacement for $\theta = 0°$ for different normal loads. (b) $\theta = 5°$ (c) $\theta = 15°$ (d) $\theta = 30°$ (e) $\theta = 45°$

Figure S6 presents simulation data of frictional behavior between micropillar surfaces with a periodicity ratio $\lambda = 1$, under five different normal loads and at various misorientation angles:

$\theta = 0°, \theta = 5°,\ \theta = 15°,\ \theta = 30°, \theta = 45°$. Figures S6 (a)-(e) show friction stress as a function of shear displacement at each misorientation. The simulations capture key trends observed in experiments as shown in videos SV4 to SV9.

S3.2 Simulation for ($\lambda = 1.023$)

Figure S7 shows simulation results of interfacial dislocation patterns between micropillar samples with a periodicity ratio $\lambda = 1.023$ across a series of misorientation angles $\theta = 0°, \theta = 5°,\ \theta = 15°,\ \theta = 30°, \theta = 45°$. Figures S7 (a)-(e) correspond to increasing misorientation: (a) $\theta = 0°$ (b) $\theta = 5°$ (c) $\theta = 15°$ (d) $\theta = 30°$ (e) $\theta = 45°$. Compared to the case of $\lambda = 1$, the slight mismatch in periodicity introduces mismatch at the interface. As θ increases, the interfacial dislocations become denser combining the effects of angular misalignment and periodicity mismatch. These simulated results are consistent with experimental observations, both in the emergence of dislocation structures and their dependence on geometric parameters.

Figure S8 shows simulated friction data for micropillar interfaces with a periodicity ratio $\lambda = 1.023$ across misorientation angles $\theta = 0°, 5°, 15°, 30°$ & $45°$, under five different normal loads. Figure S8 (a)-(e) display friction stress versus shear displacement for each misorientation. Force-displacement curves show

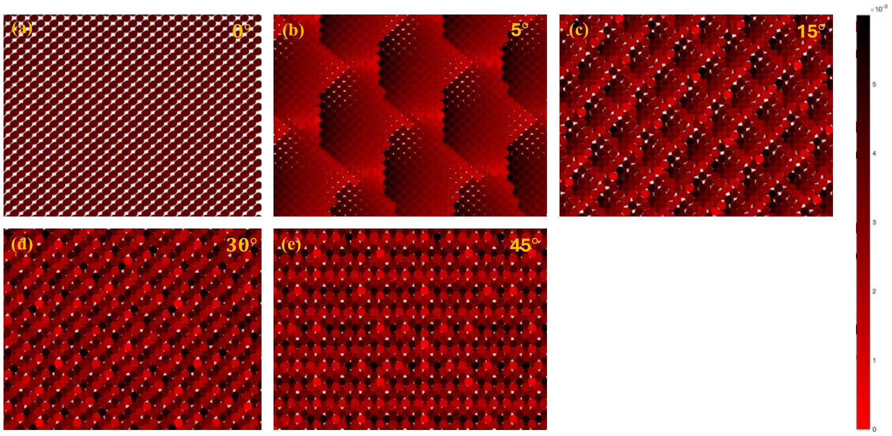

Figure S7 Interfacial dislocations between micropillar samples at $\lambda = 1.023$ at different misorientations. $a)\ \theta = 0°, b)\ \theta = 5°, c)\ \theta = 15°, d)\ \theta = 30°, (e)\ \theta = 45°$.

friction increasing with normal load. There is a weak dependence on misorientation as also seen in the experiments.

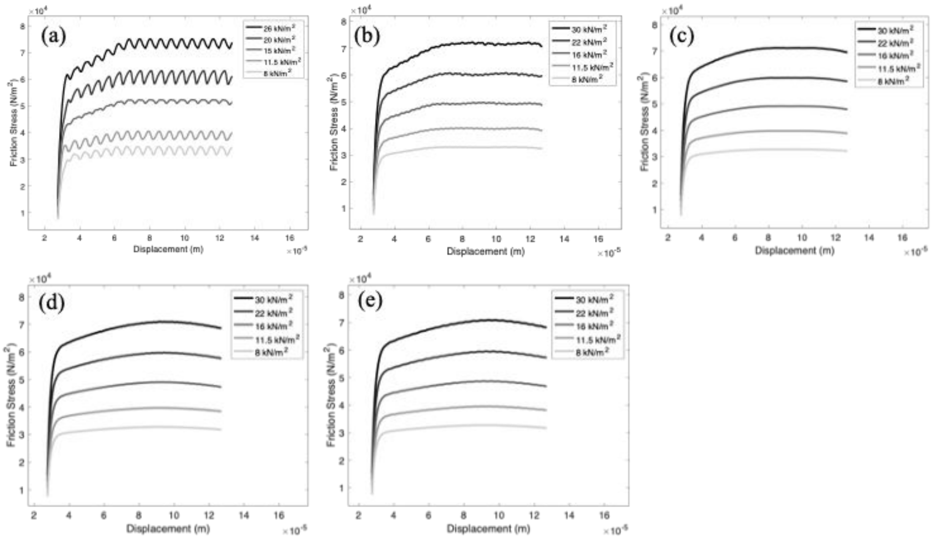

Figure S8 This figure represents friction data for $\lambda = 1.023$ at various misorientations $\theta = 0°, 5°, 15°, 30°$ & $\theta = 45°$ and five different normal loads. (a) Friction stress vs shear displacement for $\theta = 0°$ for different normal loads. (b) $\theta = 5°$ (c) $\theta = 15°$ (d) $\theta = 30°$ (e) $\theta = 45°$.

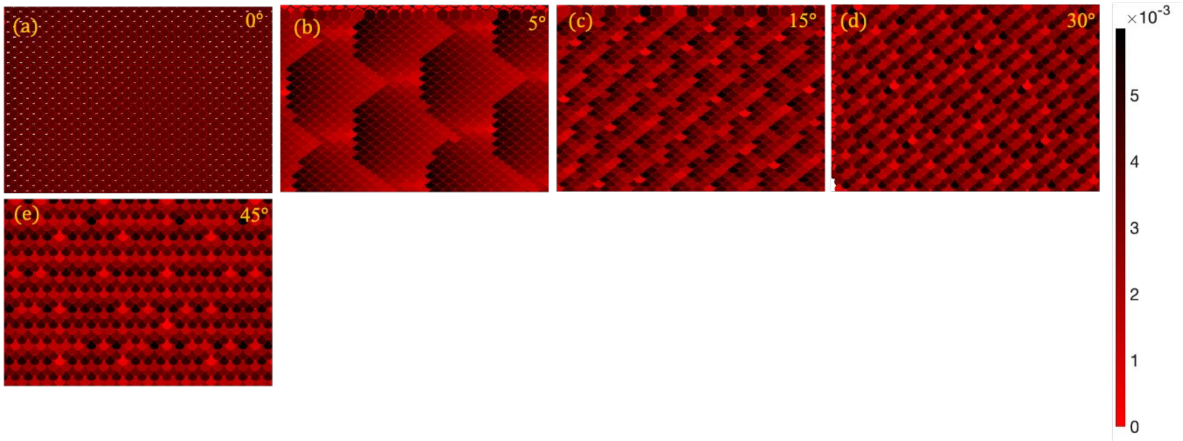

Figure S9 Interfacial dislocations between micropillar samples at $\lambda = 1.006$ at different misorientations. $a)$ $\theta = 0°$, $b)$ $\theta = 5°$, $c)$ $\theta = 15°$, $d)$ $\theta = 30°$, (e) $\theta = 45°$.

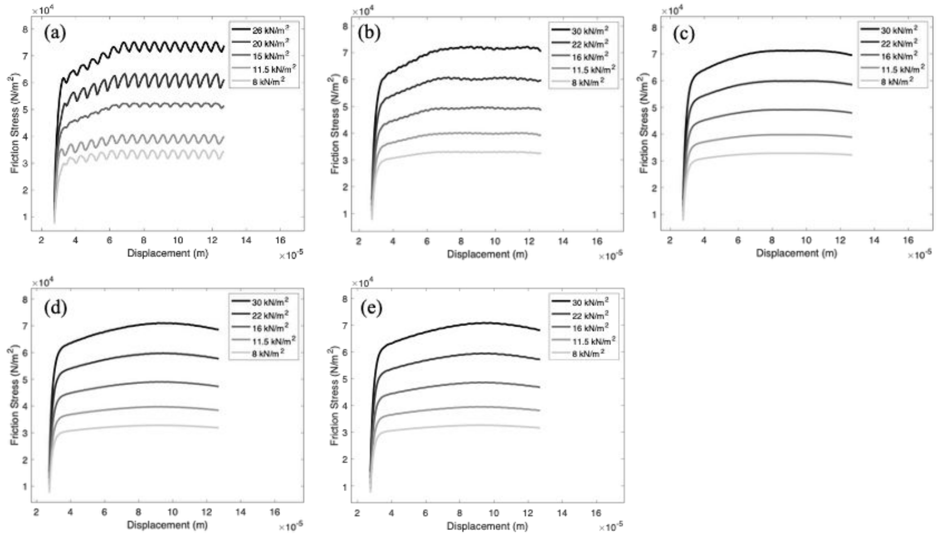

Figure S10. This figure represents friction data for $\lambda = 1.006$ at various misorientations $\theta = 0°, 5°, 15°, 30°$ & $\theta = 45°$ and five different normal loads. (a) Friction stress vs shear displacement for $\theta = 0°$ for different normal loads. (b) $\theta = 5°$ (c) $\theta = 15°$ (d) $\theta = 30°$ (e) $\theta = 45°$.

Figure S10 shows simulated friction data for micropillar interfaces with a periodicity ratio $\lambda = 1.006$ across misorientation angles $\theta = 0°, 5°, 15°, 30°$ & $45°$, under five different normal loads. Figure S10 (a)-(e) display friction stress versus shear displacement for each misorientation. Force-displacement curves show friction increasing with normal load. There is a weak dependence on misorientation as also seen in the experiments. Video SV.1. 30by30w40by30_0deg_1006.avi corresponds to friction data shown in figure S10(a).

S4. FE simulation using ABAQUS

S4.1. FE results without removing spikes

Here, we present the reaction shear and compressive forces from the pillar sliding simulations without removing the spikes. The models are the same as those used in the main manuscript, with a friction coefficient of 0.4, pillar radius $R = 1.5$ mm, and pillar height $h = 4.8$ mm. In the simulation, a shear modulus of *G*=1 MPa was used. The forces shown are scaled by a factor of 0.65 to match the experimentally fitted modulus. The bottom part consists of a 5×5 pillar array on top of a fixed substrate with dimensions 30 mm $\times$ 30 mm $\times$ 12 mm. The top part contains a single pillar at its center, supported by a smaller substrate

measuring 12 mm × 12 mm × 12 mm. The top surface of the top part is constrained in height and moves along a diagonal path across the bottom part. Figures S11, S12 and S13 show the results for $h_c = 1.2$ mm, $h_c = 2.4$ mm, $h_c = 3.6$ mm respectively.

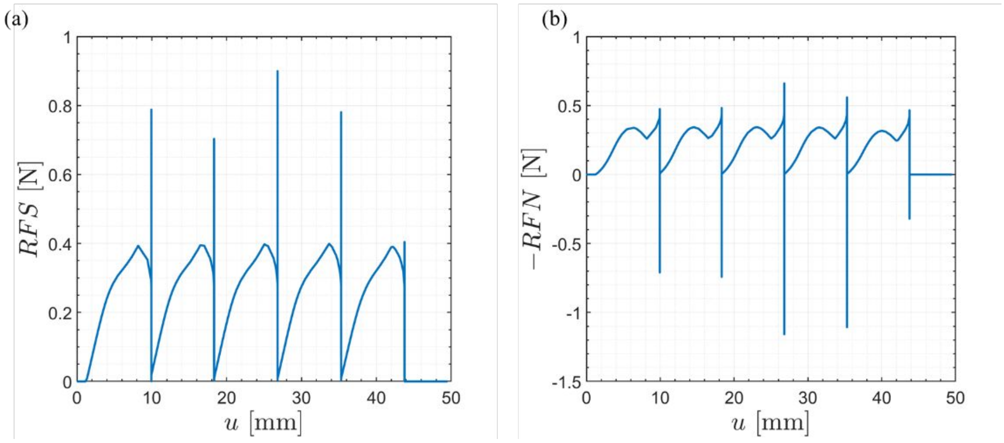

Figure S11. Raw data of reaction shear force (RFS) and normal force (RFN) on the top surface of the top part for $h_c = 1.2$ mm. In the simulation, a shear modulus of $G = 1$ MPa was used. The forces shown here are scaled by a factor of 0.65 to match the experimentally fitted modulus.

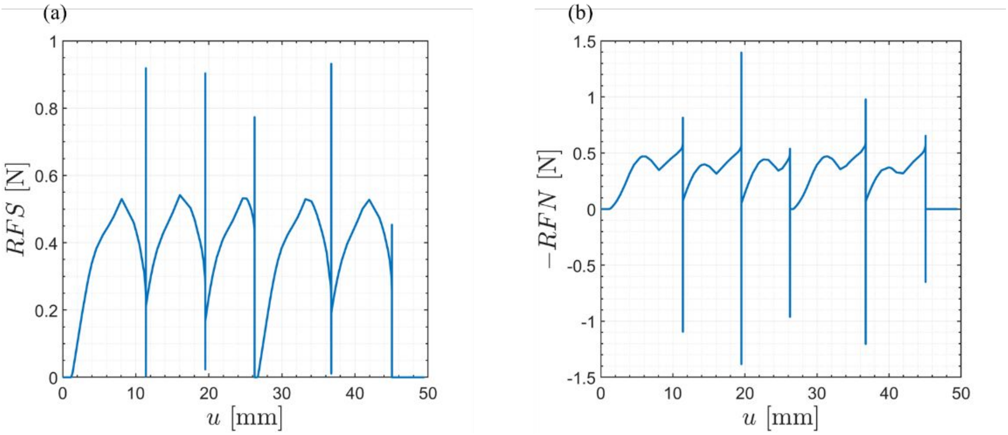

Figure S12. Raw data of reaction shear force (RFS) and normal force (RFN) on the top surface of the top part for $h_c = 2.4$ mm. In the simulation, a shear modulus of G=1 MPa was used. The forces shown here are scaled by a factor of 0.65 to match the experimentally fitted modulus.

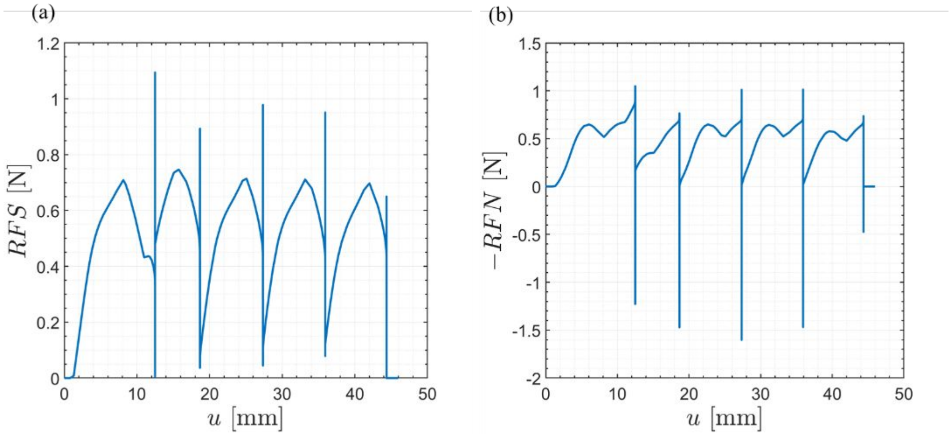

Figure S13. Raw data of reaction shear force (RFS) and normal force (RFN) on the top surface of the top part for $h_c = 3.6$ mm. In the simulation, a shear modulus of G=1 MPa was used. The forces shown here are scaled by a factor of 0.65 to match the experimentally fitted modulus.

S4.2. Mesh used for $h_c = 1.2$ mm case

As mentioned in the main manuscript, a denser mesh was used for the pillar on the top part in the $h_c = 1.2$ mm case to ensure convergence. The geometry and mesh for this case are shown in Figure S14. Figure S14(a) displays the top and front views of the mesh for the bottom part, which is the same as that used in the $h_c = 2.4$ mm and $h_c = 3.6$ mm cases. Figure S14(b) shows the mesh for the top part, where a finer mesh of 0.1 mm is applied at the edge of the pillar and along its height, transitioning to 0.2 mm toward the pillar center. Correspondingly, the in-plane mesh on the substrate is denser near the pillar edge compared to the $h_c = 2.4$ mm and $h_c = 3.6$ mm cases.

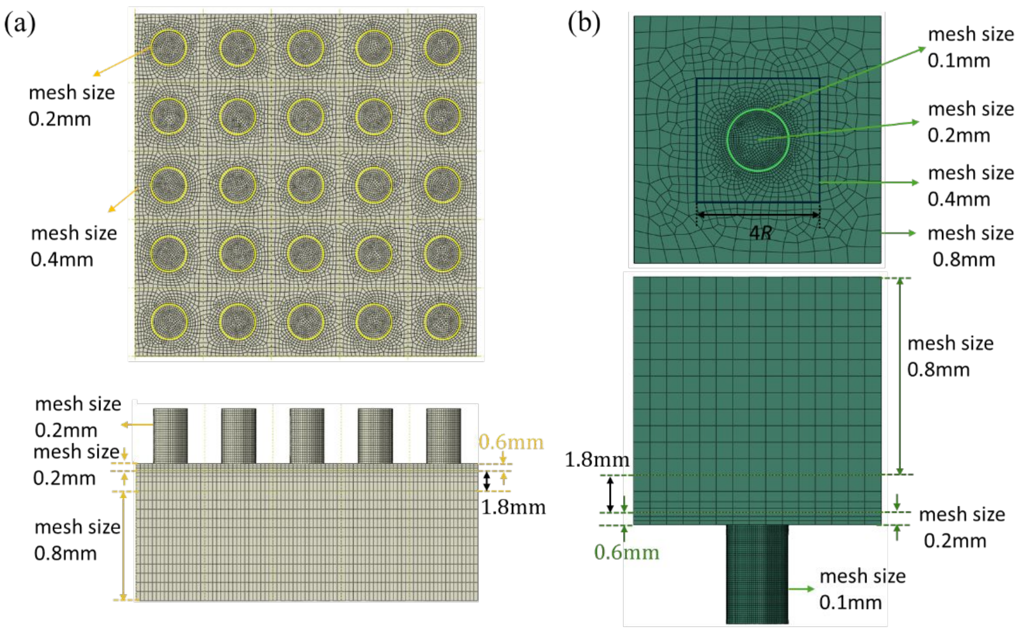

Figure S14. Mesh used for the h_c = 1.2 mm case. (a) Mesh for the bottom part, with pillars highlighted by yellow circles. (b) Mesh for the top part, with the pillar highlighted in bright green. Note that the scale differs between (a) and (b); the top part is enlarged to show mesh details.

S4.3. Mesh convergence test

To verify the accuracy of our simulation results with respect to mesh resolution, we performed a mesh convergence study using different mesh sizes. To reduce computational costs, we constructed a smaller representative model. As shown in Figure S16, this model includes only two pillars (colored dark blue) with diameter D=3mm and height of h=4.8 mm on the bottom part. These two pillars are placed diagonally, with a center-to-center spacing of 6 mm in both the x and y directions. The midpoint between their centers coincides with the center of the substrate, ensuring a symmetric layout. The substrate of the top part measures 12 mm $\times$ 12 mm $\times$ 9.6 mm, and the bottom part has an identical substrate, with a single pillar located at the center (colored dark orange). Both parts use the same material properties as in main simulations, modeled as incompressible neo-Hookean solids with a shear modulus of μ_0 = 1 MPa and a

density of $\rho = 9.65 \times 10^{-10}$ tonne/mm^3[3]. Incompressibility is enforced by setting $D_1 = 0.001$ in the material model.

The bottom surface of the bottom part is fully fixed, while the top surface of the top part is constrained in the vertical direction and moves at a velocity of $v_x = v_y = 0.5$ mm/s, i.e., diagonally across the two bottom pillars.

Although this model is smaller than that used in the main simulations containing 5×5 pillars on bottom part, it is representative for validating mesh convergence. It preserves key geometric and physical features, including the pillar spacing, pillar dimensions, and—most importantly—the contact interactions between the top pillar and the diagonally adjacent bottom pillars.

Three mesh sizes are defined in the model. Let s_1 (mm) denote the mesh size used for the pillars (both radially and axially), s_2 (mm) the in-plane mesh size at the substrate edges of the bottom part, and s_3 (mm) the in-plane mesh size at the substrate edges of the top part. In the height direction, the mesh size is set to s_1 for the first 1.2 mm from the from the interface between the pillars and the substrate, transitions to s_3 over the next 3.6 mm and remains at s_3 mm until the far end of the substrate.

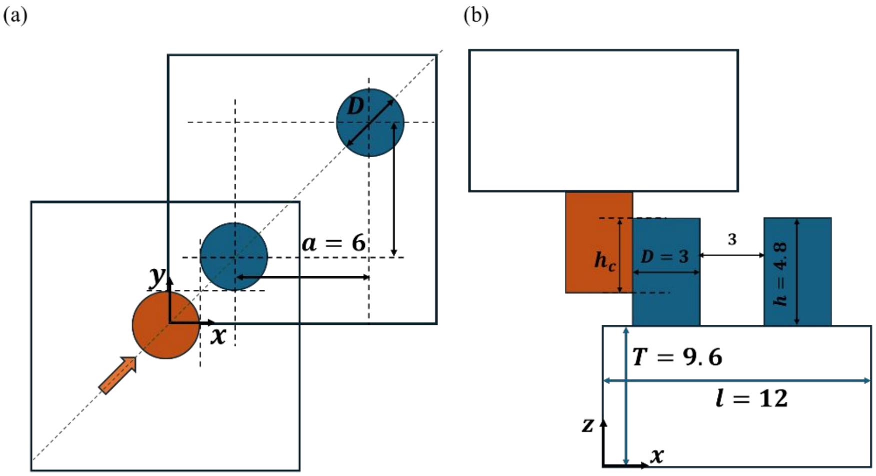

Figure S15. Geometry of the model used for mesh convergence test. The bottom part contains two pillars (colored dark blue) positioned diagonally with a center-to-center distance of $a = 6$ mm in both x and y directions. The pillars are symmetrically placed such that the midpoint between their centers coincides with the center of the substrate. The top part contains a single centrally located pillar (colored dark orange). All pillars have a diameter $D = 3$ mm and height $h = 4.8$ mm. The substrates on both parts have a length and width $l = 12$ mm and a thickness $T = 9.6$ mm.

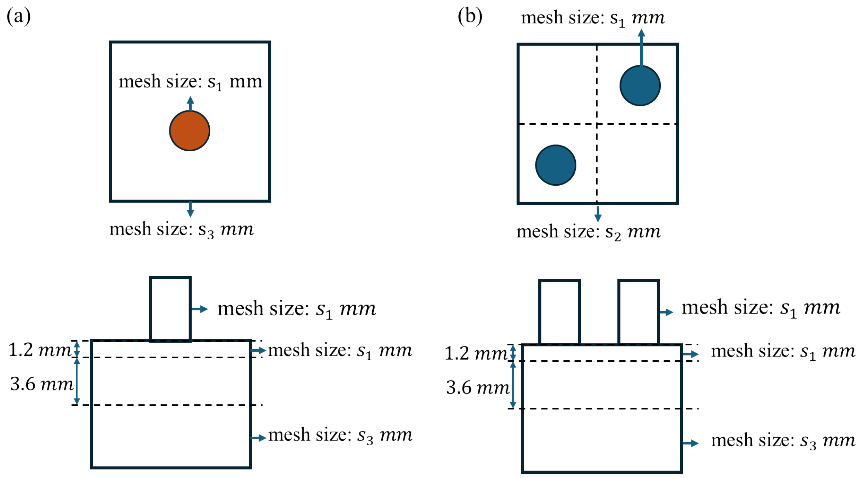

Figure S16. Mesh of the model used for the mesh convergence test. (a) shows the mesh for top part, (b) shows the mesh for the bottom part. The mesh sizes are defined as follows: s_1 is the mesh size used for all pillars (both radially and axially); s_2 is the in-plane mesh size at the substrate edges of the bottom part and along the dashed centerlines shown in the top view of (b); and s_3 is the in-plane mesh size on the substrate edges of the top part. Along the thickness direction, the mesh size is set to s_1 for the first 1.2 mm from the top surface of the substrate (i.e., the pillar base), gradually transitions to s_3 over the next 3.6 mm, and remains at s_3 until the bottom of the substrate.

For the height overlap between the two sets of pillars $h_c = 3.6$ mm, we tested five sets of mesh resolutions:

(1) $s_1 = 0.3$, $s_2 = 0.6$, $s_3 = 1.2$;

(2) $s_1 = 0.2$, $s_2 = 0.6$, $s_3 = 1.2$;

(3) $s_1 = 0.2$, $s_2 = 0.4$, $s_3 = 0.8$;

(4) $s_1 = 0.15$, $s_2 = 0.6$, $s_3 = 1.2$;

(5) $s_1 = 0.15$, $s_2 = 0.3$, $s_3 = 0.6$.

As shown in Figure S17, once the mesh becomes finer than $s_1 = 0.3$, $s_2 = 0.6$, $s_3 = 1.2$, the simulated shear force (RFS) and normal compression force (-RFN) show good consistency, particularly in terms of the peak values—excluding the spikes. Note that the forces are plotted directly from simulations with G=1 MPa, without scaling.

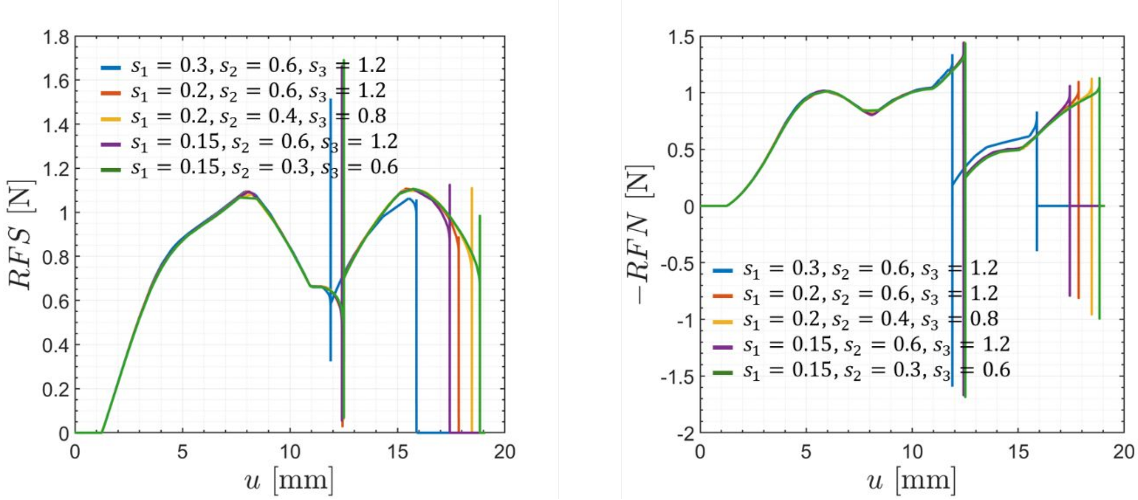

Figure S17. Reaction shear force (RFS) and compression force (-RFN) for mesh convergence test with *G*=1 MPa, $h_c = 3.6$ mm.

S4.4. Check linearity of the system

In this section, we validate the linearity of the problem by demonstrating that the reaction forces scale linearly with the shear modulus. To do this, we use the mesh convergence model with mesh parameters $s_1 = 0.2$, $s_2 = 0.4$, $s_3 = 0.8$, and introduce an additional case where the material properties are manually set with a shear modulus *G*=0.65 MPa and $D_1 = 0.0015$ to maintain near-incompressibility. Figure S19 presents a comparison between the results obtained from the scaled case (based on *G*=1 MPa) and those computed directly with *G*=0.65 MPa. The two sets of results align well, confirming the linear relationship.

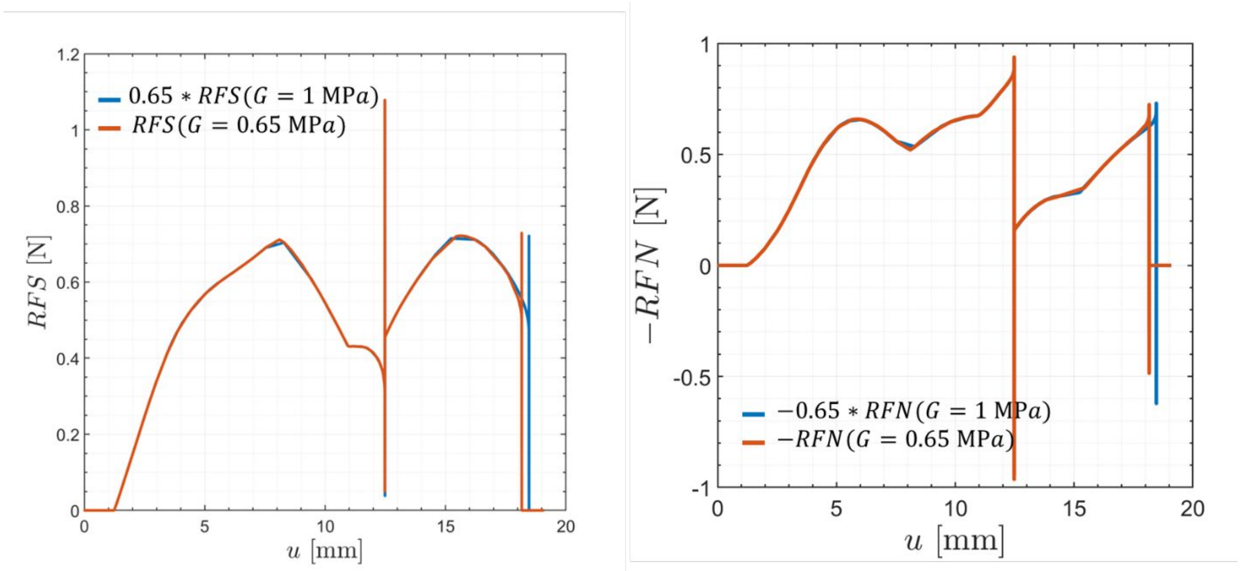

Figure S18. Validation of linearity with respect to shear modulus. Reaction shear force (RFS) and compression force (-RFN) on the top surface of the top part are compared between simulations using shear modulus G = 1 MPa (with post-scaling by 0.65) and G = 0.65 MPa (direct input).

S5. Videos

SV1_30by30w40by30_0deg_1006.avi: Sliding of 30×30 (~1770) pillars on 40×40 (~3160) array at 0° orientation, θ and λ = 1.006, in layer-based simulation.

SV2_30by30w40by40_5deg_1006.avi: Sliding of 30×30 (~1770) pillars on 40×40 (~3160) array at 5° orientation, θ and λ = 1.006, in layer-based simulation.

SV3_30by30w40by40_15deg_1006.avi: Sliding of 30×30 (~1770) pillars on 40×40 (~3160) array at 15° orientation, θ and λ = 1.006, in layer-based simulation.

SV4_10by10with20by20_0deg_2MPa.avi: Sliding of 10×10 (~190) pillars on 20×20 (~728) array at 0° orientation at stiffness 2 MPa, in layer-based simulation.

SV5_10by10with20by20_200kpa.avi: Sliding of 10×10 (~190) pillars on 20×20 (~728) array at 0° orientation at stiffness 200 kPa, in layer-based simulation.

SV6_10by10w20by20at5deg.avi: Sliding of 10×10 (~190) pillars on 20×20 (~728) array at 5° orientation at stiffness 2 MPa, in layer-based simulation.

SV7_10by10w20by20_15deg.avi: Sliding of 10×10 (~190) pillars on 20×20 (~728) array at 15° orientation at stiffness 2 MPa, in layer-based simulation.

SV8_10by10w20by20_30deg.avi: Sliding of 10×10 (~190) pillars on 20×20 (~728) array at 30° orientation at stiffness 2 MPa, in layer-based simulation.

SV9_10by10w20by20_45deg.avi: Sliding of 10×10 (~190) pillars on 20×20 (~728) array at 45° orientation at stiffness 2 MPa, in layer-based simulation.

SV10_10by10w20by20_0deg.avi: Sliding of 10×10 (~190) pillars on 20×20 (~728) array at 0° orientation at stiffness 2 MPa, in layer-based simulation. Recording with the corresponding force plot.

SV11_10by10w20by20_0deg_200Kpa.avi: Sliding of 10×10 (~190) pillars on 20×20 (~728) array at 0° orientation at stiffness 200 kPa, in layer-based simulation. Recording with the corresponding force plot.

SV12_60-RT_0_deg_5x_Nov13_(-1V)_1.avi: Sliding friction experiment at θ = 0°, 5x magnification, −1 V normal load, λ = 1.006.

SV13_60-RT_5_deg_5x_Nov23_(-0.5V)_2.avi: Sliding friction experiment at θ = 5°, 5x magnification, −0.5 V normal load, λ = 1.006.

SV14_60-RT_15_deg_5x_Nov23_(-0.5V)_1.avi: Sliding friction experiment at θ = 15°, 5x magnification, −0.5 V normal load, λ = 1.006.

SV15_60-RT_30_deg_5x_(-1.5V)_1.avi: Sliding friction experiment at θ = 30°, 5x magnification, −0.5 V normal load, λ = 1.006.

SV16_60-RT_45_deg_5x_(-0.5V)_1.avi: Sliding friction experiment at $\theta = 45°$, 5x magnification, −0.5 V normal load, $\lambda = 1.006$.

SV17_RT-RT_0_deg_5x_Nov9_(-0.5V).avi: Sliding friction experiment of pillar-pillar interface at $\theta = 0°$, 5x magnification, −0.5 V normal load, $\lambda = 1$.

SV18_RT-RT_5_deg_5x_Nov9_(-0.5V).avi: Sliding friction experiment of pillar-pillar interface at $\theta = 5°$, 5x magnification, −0.5 V normal load, $\lambda = 1$.

S6. References

[1] J. Dillen, Z. He, C.-Y. Hui, and A. Jagota, "Geometry of defects at shape-complementary soft interfaces," *Extreme Mechanics Letters,* vol. 9, pp. 74-83, 2016, doi: 10.1016/j.eml.2016.05.006.

[2] K. L. Johnson, *Contact Mechanics*. Cambridge: Cambridge University Press, 1985.

[3] S. K. Patel, S. Malone, C. Cohen, J. R. Gillmor, and R. H. Colby, "Elastic modulus and equilibrium swelling of poly(dimethylsiloxane) networks," *Macromolecules,* vol. 25, no. 20, pp. 5241-5251, 1992/09/01 1992, doi: 10.1021/ma00046a021.
